# Adjudicating Credibility: Documenting the Role of Mental Health Immigration Forensic Assessments

**DOI:** 10.1017/amj.2025.10066

**Published:** 2025-07

**Authors:** Alea Skwara, Sharon Howard, Carmen Velazquez, Raquel Aldana

**Affiliations:** UC Davis School of Law, Davis, CA USA

**Keywords:** immigration forensic evaluations, adjudicating credibility in immigration proceedings, law and the science of trauma

## Abstract

Mental health or psychological forensic assessments are a growing practice in immigration adjudication, but the practice is not well understood. Several studies have measured the impact of medical or mental health forensic reports in immigration adjudication; yet none have documented when mental health forensic reports are sought or how they are conducted in practice. This article undertakes an interdisciplinary empirical documentation of the practice of forensic mental health assessments in immigration adjudication. A core focus of our survey was documenting the role of mental health forensic immigration assessments in substantiating migrants’ trauma and bolstering credibility. Our preliminary findings identify ways to improve the practice of mental health assessments within the immigration context toward practices that are more consistent with the science of trauma and memory.

In a recent publication titled *Trauma as Inclusion*, we documented the evolution of U.S. immigration law from treating trauma solely as a ground for exclusion to recognizing its occurrence as a potential for inclusion.^1^ Today, it is possible on a few narrow grounds that past or future trauma — whether based on persecution, crime, or child abuse or abandonment — can become the basis for migrants to seek a path to immigration legalization.^2^ In a few instances, the psychological harms emanating from border enforcement — such as family separation — can also lead to relief against deportation.^3^ And because law treats these immigration benefits or relief as an exercise in humanitarian grace — and thus largely discretionary — the onus is on the migrant to petition for the remedy affirmatively, or as relief from removal, and to convince the adjudicator that they are deserving.^4^

Deservedness in securing a humanitarian visa or relief against deportation often turns on whether a migrant’s documentation of past or the risk of future trauma is credible.^5^ To establish credibility, migrants must attempt to document their trauma, including by narrating their own stories, which they must tell several times under stressful and often adversarial circumstances.^6^ Yet, legal and scientific understandings of credible narrations of trauma diverge significantly. Immigration legal norms and practices expect migrants to speak of their trauma chronologically and with consistency, detailing facts that might suggest an impeccable memory and with a certain demeanor whose contours vary according to the adjudicator’s own biases.^7^ In contrast, psychological and neuroscience research demonstrates that autobiographical memory — memory for the events of one’s own life — is disjointed and fragmented.^8^ Thus, while adjudicators expect a linear and coherent narrative to lend credibility to the cases they see, this expectation is inconsistent with current scientific understandings of memory.^9^ This is exacerbated in instances of trauma, where the very brain systems required to form and recall autobiographical memories are compromised,^10^ leading to even more fragmented, sensorily-based, and potentially inconsistent narratives.^11^ In children, memory and cognition — the ability to recall and recount a memory in a coherent fashion — are dependent on their development as well as the types and frequency of early trauma and social context.^12^ Yet, immigration law does not always apply different procedures, burdens, or standards to the adjudication of immigration cases involving children.^13^

To bridge this legal-scientific gap in trauma-based adjudication of credibility, immigration lawyers, on behalf of their clients, have turned to mental health providers and medical doctors to provide forensic assessments to help bolster claims of trauma. Studies show that the submission of medical immigration forensic evidence in the adjudication of immigration cases that require proof of trauma substantially improves the odds of securing the immigration benefit.^14^ Medical forensic reports are especially effective when physical traces of past trauma are evident in the body of the person seeking immigration protections, such as in asylum cases. But physical traces of trauma are not always available. Physical traumas — even instances of torture or sexual assault — do not always leave scars or permanent physical traces that can be documented through a physical exam.^15^ Documentation of psychological harm presents an even more complicated case: psychological traumas, even when they have a physiological impact, cannot easily be traced in the body, or at least not in ways that easily translate into valid legal forensic proof of their occurrence.^16^ When this happens, mental health — rather than medical immigration forensic assessments — may be the sole option for introducing forensic documentation of trauma.

Mental health forensic assessments do not physically verify past trauma; however, they can document whether a person’s present psychological condition, including as it pertains to any gaps in memory, is consistent with past trauma.^17^ There are several psychological tests and assessment tools, for example, designed to measure trauma exposure and its impact.^18^ These psychological instruments can be incredibly useful to corroborate trauma, although they do not always adequately account for cultural differences^19^ or capture individual differences in the responses to trauma.^20^ Importantly, not every person who endures even severe forms of trauma will develop post-traumatic stress disorder (PTSD),^21^ which is too often treated as a proxy for past trauma. For example, psychological studies have shown that persons who grew up in loving and stable households develop greater resiliency to adversity that could help them overcome mental health distress from trauma.^22^ As such, it is essential that any approach to adjudicating trauma considers how cultural and individual differences may influence clinical presentation. Trained mental health providers can help explain this important science to advocates representing trauma survivors and the decision-makers adjudicating this trauma. Mental health experts can also help document how anticipated trauma — such as the prospect of family separation from immigration removal — is impacting the mental health of family members who would be left behind in the United States.^23^ Thus, mental health immigration forensic assessments represent an important opportunity in immigration cases to educate decision-makers on scientific perspectives of what would constitute a credible story, as well as explain the impact of trauma on the petitioner’s ability to tell their story.

While there are studies measuring the impact of medical or mental health forensic reports in immigration adjudication,^24^ no studies have documented when mental health forensic reports are sought or how they are conducted in practice. This is especially important given that immigration forensic assessments are a growing practice in immigration adjudication in the United States and yet existing norms do not prescribe practice guidelines for the evaluation of immigration populations as part of legal proceedings.^25^ The Istanbul Protocol on the Effective Investigation and Documentation of Torture and Other Cruel, Inhuman or Degrading Treatment or Punishment, first adopted in 2001, sets out international standards on how effective legal and medico-legal investigations into allegations of torture or ill-treatment should be conducted.^26^ The Istanbul Protocol is widely recognized as the gold standard for documenting torture and ill-treatment in legal cases that call for the evaluation of past trauma.^27^ However, the Istanbul Protocol is not binding for U.S. immigration adjudicators who are not trained in its guiding principles and best practices.^28^ These norms and other best practices are employed by medical and other professionals and incorporated in training or curricula across numerous medical and social work schools.^29^ However, it is not well documented whether and how these norms are incorporated in practice. Our survey of immigration lawyers and mental health providers^30^ who procure or conduct mental health immigration forensic assessments is a first effort to document and start to understand the practice as it occurs in immigration adjudication.

A core focus of our survey was documenting the role of mental health forensic immigration assessments in substantiating migrants’ trauma and bolstering credibility. In addition, we attempt to document the degree of inter-professional consensus of key terms and differences that pertain to issues of credibility (or related terms such as malingering) and whether lawyers, adjudicators and evaluators have a different understanding of what credibility means and the factors that are relevant to its determination. Relatedly, we try to document the understandings of “trauma” across disciplines, as well as how credibility is assessed across professions and how the issue is reported, if at all, in immigration forensic reports. Finally, to the extent that these differences exist, we also sought to learn what concerns, if any, about these differences exist across professions. Are there practices that should be avoided in immigration forensic reports that relate to the assessment of credibility? Our study provides insights into how immigration forensic reports are being conducted to help instruct immigration lawyers and mental health providers as they consider how to improve the practice. Below is a discussion of our study and the reporting of our key findings about the role of trauma and mental health forensic immigration assessments in credibility determination.

## I. Methodology

To conduct our study of the practice of mental health immigration forensic assessments, we convened an interdisciplinary team composed of researchers and practitioners in law, psychology, neuropsychology, neuroscience, and psychiatry with experience in immigration law, or the science of trauma, or both.^31^ Over several months, we designed two distinct survey instruments together — one for lawyers and one for mental health providers — with parallel questions that could allow for comparison across surveys. We recruited participants through posts on social media,^32^ relevant professional listservs,^33^ and targeted outreach. The targeted outreach included hundreds of lawyers and mental health practitioners in our teams’ professional networks, including stakeholders working with refugee communities in the greater Sacramento and Bay areas who were part of a working group formed in our earlier efforts to bring together lawyers and mental health professionals to discuss matters relevant to refugee mental health.^34^ It also included immigration lawyers who are members of the American Immigration Lawyers Association (AILA) and who practice in states with large concentrations of immigrants and with a presence of immigration courts.^35^ There is currently no professional organization for mental health practitioners who practice immigration forensics. Therefore, our outreach efforts to mental health professionals relied on related professional organizations, such as the Center for Gender and Refugee Studies — which we knew worked to connect immigration lawyers with mental health providers — to request that they post our survey in their newsletters or websites. We also asked immigration lawyers to forward the mental health survey to mental health practitioners with whom they work and relied on our own professional networks for word-of-mouth publicity. Aside from our outreach through social media and listservs, we sent emails to approximately 600 immigration lawyers and approximately 200 mental health professionals. These numbers represent our best estimate of survey reach, as the exact number of recipients on our targeted listservs and the number of individuals who viewed our social media posts is unknown. All study procedures were approved by the University of California, Davis Institutional Review Board.

We received usable responses from ninety-six lawyers and fifty-two mental health providers, including partial, but usable, data. Of these, seventy-six lawyers and thirty-seven mental health providers fully completed the survey instrument, including answering the demographic questions asked at the end of the survey. As different questions have different numbers of respondents due to this attrition, the total (N) for each question is provided when we report results.

## II. Study Findings and Implications

### A. Survey Respondents

We asked survey respondents to complete the survey only if they currently or have previously conducted or used mental health immigration forensic assessments as part of their mental health or legal practice. Legal professionals were primarily licensed attorneys who represent immigrants in immigration proceedings, but also included other legal professionals who are not lawyers but who may be authorized under state or federal law to provide some legal-related services to immigrants.^36^ Ninety-eight percent of legal professionals (sixty-nine out of seventy-five respondents) reported being licensed to practice law, while eight percent (six respondents) reported that they were not. Consistent with our recruitment strategy, the states in which ten percent or more of licensed respondents reported holding a license were California (41.18%), New York (26.47%), Florida (10.29%), and Texas (10.29%).

The mental health survey was open to any mental health practitioner who has conducted at least one immigration forensic assessment. This flexible approach was necessitated by the wide variation in the types of degrees and training held by mental health professionals who provide forensic immigration assessments — from psychologists and psychiatrists to family therapists and social workers. This variation is due in part to the relative newness of this area and in part to the fact that few mental health practitioners are engaging in this kind of work. In contrast to the common or even mandatory use of forensic assessments in other areas of the law, such as tort^37^ or criminal law,^38^ the use of immigration forensic assessments in immigration proceedings is an emerging field that is neither required under legal norms nor easily accessible to most immigrants. In general, the practice has grown in recent years based on efforts by immigration advocates who see the need for infusing science-based understandings to the adjudication of trauma and credibility in immigration proceedings.^39^ Yet, the lack of market-based monetary incentives (such as ones that exist in tort law) or public funding (such as in criminal cases) to pay for immigration forensic assessments has meant that few mental health practitioners can afford to provide them since many immigrants must seek these services with little or no pay.

In our survey, mental health practitioners reported practicing with a range of professional degrees and licenses. An equal number of respondents (N=37) reported practicing as clinical psychologists (PhD/PsyD) or as licensed clinical social workers (LCSW), both at 35.14%. Another 10.81% reported working as licensed professional counselors (LPC), while only 5.41% and 2.70% reported working as licensed marriage and family therapists (LMFT) or as psychiatrists (MD), respectively. Another 13.51% reported that they held other degrees or licenses not listed. This variation in skills and training may be an important factor to consider when examining differences in conducting and reporting immigration forensic assessments. It is also important to consider curriculum reform and training in each of these fields of study to strengthen capacity. The Istanbul Protocol does not require forensic expert certification and recognizes that various professionals such as nurses and social workers can be trained and equipped to conduct mental health forensic assessments.^40^ It also requires that all clinicians who conduct clinical evaluations of alleged or suspected cases of torture or ill-treatment should do so in accordance with the Istanbul Protocol.^41^

In the demographic questions for both “lawyers and legal professionals” and “mental health practitioners,” we sought to understand their racial and ethnic composition, as well as their linguistic diversity, to better comprehend how their demographics might compare to the migrant populations they serve. For all demographic questions, respondents were allowed to select all that applied as categories were not mutually exclusive. The premise of our question relates to studies, in both psychology and law, linking culturally-responsive practices to quality representation or services.^42^ For example, sex and gender differences should guide assessments because they affect how persons experience and respond to trauma.^43^ We do not assume that the sex and gender identity of the professionals dictate their awareness or training on these sex and gender differences, but we do know that shared sex and gender experience may be relevant to establishing trust between client and provider.^44^ Yet, we found little sex and gender diversity among both legal and mental health professionals. Participants were allowed to select all gender identities that applied to them. In terms of the gender identity of “immigration lawyers or legal professionals” (N=76), the vast majority identify as women (77.63%), with only 18.42% identifying as men. The gender identity of mental health professionals (N=42) was similar, with 81.08% identifying as women and 21.62% identifying as men. Very few “immigration lawyers or legal professionals” or “mental health providers” working in this field reported being non-binary (3.95% and 2.7%, respectively); gender fluid (1.32% and 2.7%, respectively); genderqueer (1.32% and 0%, respectively); questioning or unsure (1.32% or 0%, respectively); or transgender (1.32% and 2.7%, respectively).

Not unlike gender, divergent lived experiences across race or ethnic differences may also yield different responses to trauma.^45^ Increasing the proportion of racial diversity among mental health providers could improve trust between patient and provider, including by yielding more culturally appropriate treatment.^46^ Additionally, racially-aware intra- and inter-racial lawyering is necessary to improve legal advocacy^47^ while ethnic and legal diversity in the profession can also potentially help reduce racial or ethnic bias in legal systems.^48^ Our survey revealed a significant lack of racial and ethnic diversity among legal and mental health professionals. This does not *per se* negate a lack of racial or ethnic consciousness among professionals; it does, however, raise questions on whether bias in the representation, assessment, or adjudication is impacted by divergence in the racial and ethnic composition between clients and the legal or mental health professional. In terms of race and ethnic background (once again, survey respondents were allowed to select all that applied), a large majority of both “immigration lawyers or legal professionals” (N=76) and “mental health practitioners” (N=37) reported being white (61.84% and 72.97%, respectively), while Hispanic or Latinx was the second largest group represented for both professionals (27.63% and 24.32%, respectively). Furthermore, while many other racial or ethnic categories were selected for both “immigration lawyers or legal professionals” and “mental health practitioners,” the representation for each of these categories was extremely low: Middle Eastern (5.26% and 5.41%, respectively); Afro-Caribbean (3.95% and 2.7%, respectively); Black or African-American (3.95% and 2.70%, respectively); Japanese (2.63% or 0%, respectively); American Indian or Alaskan Native (1.32% or 0%, respectively); Indian (1.32% and 5.41%, respectively); Korean (1.32% and 0%, respectively); and other Asian (1.32% and 5.41%, respectively).

Language capacity matters a great deal to access and quality of services in both law and mental health. For example, language barriers can mean fewer clients seek these services.^49^ Importantly, sharing the same language between providers and clients can also improve the quality of the experience and of the services provided.^50^ Of course, interpreters, especially when skilled and utilized well to bridge cultural divides, can help improve both access and quality.^51^ Unfortunately, interpreters can also adversely impact not only the quality of the assessment or representation, but at times, even introduce error and bias.^52^ The good news in our survey is that bilingualism among respondents is high. Of the seventy-six legal professionals who provided demographic data, sixty-eight of them (89.47%) reported speaking another language in addition to English. Of these sixty-eight respondents, twenty-one (30.88%) reported speaking more than two languages. Of the thirty-seven mental health professionals who provided demographic data, twenty-six (70.27%) reported speaking another language in addition to English, with eight (21.62%) reporting speaking more than two languages. The bad news in our survey is that the languages represented in the pool are few or spoken by very few. The vast majority of “lawyers or legal professionals” (N=68) and “mental health practitioners” (N=26) who reported speaking another language other than English reported speaking Spanish (88.24% and 69.23%, respectively). Other languages reported spoken by either “lawyers or legal professionals” or “mental health practitioners” were: French (16.18% and 15.38%, respectively); Portuguese (8.82% and 3.85%, respectively); Mandarin Chinese or other Chinese dialect (5.88% and 0%, respectively); Italian (4.41% and 3.85%, respectively); Arabic (1.47% and 3.85%, respectively); Bengali, Hindi, Indonesian (all 0% and 3.85%, respectively); Japanese (2.94% and 0%, respectively); Russian, Urdu, German (all 1.47% and 3.85%, respectively); Malay, Hebrew, Peruvian, Polish (all 0% and 3.85%, respectively); and Greek, Farsi, Turkish, Estonian, Korean, Swahili, and ASL (all 1.47% and 0%, respectively). It is interesting to note that in recent years in the United States, the languages most represented among applicants — at least in terms of those who seek asylum — are Spanish, Creole or French, Dari and Pashto, and Portuguese.^53^ Of these, only Spanish was well represented in our sample.

Finally, we asked about employment settings to understand whether the type of practice was relevant to the procurement or provision of mental health forensic assessments. Once again, participants were allowed to select all that applied, as we understood that both legal and mental health professionals may simultaneously work in multiple settings and some categories overlap. We sought to understand how issues of access to mental health professionals might differ based on factors like client resources (i.e., ability to pay a private attorney) or practice resources (i.e., ability of law clinics to tap into university health programs). Our survey revealed that procurement and provision of mental health immigration forensic reports occur in both public and private settings, with legal professionals in the public sector securing slightly more. For mental health professionals, the inverse was true. Respondents reported working both in private practice and in nonprofit settings. Among immigration “lawyers or legal professionals” (N=96), 45.83% reported working in private practice while 67.7% in various nonprofit settings, including nonprofit immigration clinics (32.29%), other nonprofit organizations (19.79%), and law school immigration clinics (15.62%). However, only a few worked at a refugee resettlement agency (1.04%), for the government (1.04%), or in other sectors (4.17%).^54^ The breakdown was almost the inverse for mental health providers (N=52), the majority of whom reported working in private practice (69.23%) and for a nonprofit organization (40.38%), with only a handful in a hospital (5.77%) or in other unspecified sectors (7.69%). These data alone do not reveal how and whether access issues impact the availability of mental health immigration forensic assessments. To examine this issue, we also asked legal professionals about the frequency with which they seek mental health forensic assessments, whether they are satisfied with this frequency, and what limiting factors exist in their ability to seek these assessments. In terms of frequency, the highest proportion (20.88%) of legal professionals (N=96) indicated that they pursued them in fewer than 10% of their cases. Twelve and a half percent said they pursued them in 10% to 25% of their cases; another 15.62% reported doing so in 25% to 50% of their cases, and 17.71% said they did so in 50% to 75% of their cases. Only 4.17% of legal professionals sought them in greater than 90% of their cases (another 12.5% indicated that it depended on the client or type of case). When asked if they would ideally include assessments in a different proportion of their cases than they currently do, the majority (62.50%), indicated that they would like to include them in a greater proportion of cases, with only 30.21% reporting being satisfied with the current proportion, 3.12% indicating that they would prefer to include them in fewer cases, and 4.17% being unsure. Among attorney respondents who said they would like to include evaluations in a greater proportion of their cases (N=60), all but six cited cost as a limiting factor. Other important barriers cited in a majority of responses included the lack of availability of trained mental health evaluators and timeline restrictions of cases.

### B. Types of Immigration Cases

One goal of our survey was to better understand the types of immigration cases for which mental health forensic assessments are sought. We expected our results to correlate with the legal burdens that migrants bear to prove trauma to secure the immigration relief and the associated challenges with establishing credibility or finding corroborating evidence. We asked immigration “lawyers or legal professionals” (N=96) in what types of cases they sought mental health forensic assessments from a menu of eleven options. These options were selected for our survey because the relief for these types of cases likely require proof of trauma.^55^ We also included the category of “other” to capture case types we did not explicitly list.^56^ Respondents were allowed to select all that applied. The most frequently selected case types — with at least half of respondents indicating they have sought mental health forensic assessments for this type of case — were asylum (87.50% of respondents); Violence Against Women Act or VAWA (64.58%); U visas (62.50%); waivers of inadmissibility (56.25%); and Convention Against Torture or CAT (50%) (see Table 1). Mental health providers indicated a similar distribution of case types for which they have been asked to provide a forensic assessment. The four categories selected by half or more of the respondents were asylum (92.31%), cancellation of removal (71.15%), VAWA (71.15%), and U visa (69.23%).

**Table 1.** Types of cases in which mental health forensic immigration reports are sought, as reported by legal and mental health professionals

Legal Professional Reported Case Types			
Number of Cases = 96			
Total Response Count = 473			
Response	Frequency	Percent of Responses	Percent of Cases
Asylum	84	17.76	87.50
VAWA	62	13.11	64.58
U visas	60	12.68	62.50
Waivers of inadmissibility	54	11.42	56.25
Convention against torture	48	10.15	50.00
Withholding of deportation	45	9.51	46.88
Cancellation of removal	44	9.30	45.83
T visas	24	5.07	25.00
SIJ	17	3.59	17.71
Removal of conditional status	16	3.38	16.67
Naturalization	12	2.54	12.50
Other (please list):	7	1.48	7.29

Mental Health Professional Reported Case Types			
Number of Cases = 52			
Total Response Count = 262			
Response	Frequency	Percent of Responses	Percent of Cases
Asylum	48	18.32	92.31
Cancellation of removal	37	14.12	71.15
VAWA	37	14.12	71.15
U visas	36	13.74	69.23
T visas	24	9.16	46.15
Withholding of deportation	22	8.40	42.31
Convention against torture	16	6.11	30.77
Waivers of inadmissibility	15	5.73	28.85
Removal of conditional status	10	3.82	19.23
Other (please list)	7	2.67	13.46
Naturalization	5	1.91	9.62
SIJ	5	1.91	9.62

**Table 2.** Attorney reports of cited reasons for adverse credibility determinations.^91^

Reasons Cited for Adverse Credibility Determinations			
Number of Cases = 66			
Total Response Count = 202			
Response	Frequency	Percent of Responses	Percent of Case
Inconsistency within client story/stories	59	29.21	89.39
Inconsistency between client story and evidence	51	25.25	77.27
Client demeanor	35	17.33	53.03
Lapses in client memory	31	15.35	46.97
Inconsistency between client story and forensic evaluation	13	6.44	19.70
Other (please list):	13	6.44	19.70

Several factors could influence the frequency with which legal professionals seek mental health forensic assessments in different immigration case types. The simplest reason is variation in frequency with which legal professionals encounter a given case type in their respective immigration practices. In recent years, asylum has accounted for hundreds of thousands of new applications per year.^57^ In contrast, fewer than 5,000 T visa applications are filed per year,^58^ while U visas are filed in the tens of thousands per year.^59^ Another factor may depend on whether lawyers or legal professionals have access to other means of proving their client’s trauma or establishing credibility. For example, cases like U and T visas require law enforcement certification or cooperation to establish the underlying qualifying crime or trafficking,^60^ which could bolster both the evidence of trauma and the client’s credibility. Similarly, SIJ cases could already have a juvenile or family court order establishing the child’s abuse, abandonment, or neglect by one or both of the child’s parents,^61^ making mental health forensic assessments redundant. In contrast, an asylum case based on persecution occurring outside of the United States may lack adequate corroborating evidence,^62^ and thus rely more heavily on documenting the impact of alleged trauma. Another reason could be that some of the relief only requires evidence of trauma in exceptional cases, such as with naturalization^63^ or removal of conditional status,^64^ and so mental health assessments are generally less relevant in these cases. The issue of case type also raises the question of whether the immigration forum in which a case will be seen (USCIS vs. Immigration Court) affects the frequency with which a mental health forensic assessment is sought. While some case types are consistently seen in one forum or the other, sometimes the circumstances of a particular case — such as whether the asylum seeker entered with a visa or presented credible fear at the border — can affect the forum in which that case is heard. Therefore, to assess differences between immigration forums, we would need to ask directly whether the immigration forum affected the decision to seek an assessment, or to collect responses at the level of the individual case. As the current survey only asked about which case types respondents had ever sought an assessment for (and not about individual cases or the number of times they had sought an assessment for a given case type), the current data cannot offer any insight into the question of forum type. This also represents a limitation in the interpretation of case type frequency — the frequency in the current study represents the percentage of respondents who have sought a mental health forensic assessment in a given case type but does not give us insight into the relative frequency with which these assessments are sought for different case types.

The frequency with which lawyers or legal professionals seek mental health forensic assessments in certain types of immigration cases may also correlate with the reason they seek a mental health assessment in the first place. When asked in the survey what factors led them to seek a mental health forensic assessment in their cases, “immigration lawyers or legal professionals” (N=96) cited the need to validate past harm, cruelty, or hardship claims as the top factor (93.75% endorsed this response). Addressing credibility concerns (e.g., internal inconsistencies in the client’s statements) was the third most frequently endorsed factor (65.62%).^65^ Consistently, these same two factors ranked first (94.23% citing the need to validate past trauma, cruelty, or hardship claims) and third (55.77% citing credibility concerns) when mental health evaluators (N=52) were asked what reason lawyers give for seeking a mental health forensic assessment. The second most frequently cited reason across both “lawyers or legal professionals” (68.75%) and “mental health providers” (67.31%) was the need for a mental health diagnosis in an immigration case. This raises the question of whether lawyers consider a mental health diagnosis as legally relevant to a migrant’s experiences of trauma, and consequently, their credibility. If so, this could be problematic given scientific findings that trauma does not always result in mental health conditions such as post-traumatic stress disorder^66^ and is therefore not a reliable proxy for trauma.

### C. Types of Evaluations Sought

We also asked immigration lawyers or legal professionals what they asked evaluators to address as part of an immigration forensic assessment. Conversely, we asked mental health evaluators what they have been asked to include in their forensic evaluations by immigration lawyers or legal professionals. The surveys reveal fairly consistent responses to these questions. Respondents in both surveys, for example, identified the top five answers in the exact same order as follows: impact of trauma (lawyers or legal professionals — 97.92%; mental health professionals — 100%); general psychological/mental health evaluation of the immigration petitioner (lawyers or legal professionals — 88.54%; mental health professionals — 90.38%); a specific diagnosis for mental health (lawyers or legal professionals — 83.33%; mental health professionals — 82.69%); credibility of the immigrant story and of the immigrant (lawyers or legal professionals — 64.58%; mental health professionals — 61.54%); and consistency of overall information (lawyers or legal professionals — 56.25%; mental health professionals — 55.77%). These responses reveal the importance lawyers or legal professionals place on seeking to document the impact of trauma and their client’s credibility through mental health forensic assessments and demonstrate a high degree of interprofessional consistency. However, they also reveal the importance that lawyers place on securing a general or specific mental health diagnosis of their immigration clients. This could signal a misunderstanding on the part of the lawyer or legal professional about the correlation between trauma and mental health,^67^ or worse, expectations by the immigration adjudicator that only a mental health diagnosis can establish definitively that trauma occurred.^68^ In other words, even if the lawyer or legal professional themself understands that the absence of a mental health diagnosis does not signal the absence of trauma, they may view the diagnosis as necessary to convince the immigration adjudicator of the significance and validity of past trauma.^69^ Relevant to this topic, we discuss perceptions of immigration adjudicators’ knowledge of trauma in Part G of this Article.

Lawyers or legal professionals also reported asking evaluators to include a specific diagnosis of neurocognitive/developmental disorders (54.17%); behavioral assessment/demeanor (51.04%); rehabilitation of addiction (30.21%); and character assessment (19.79%). Once again, there was a high degree of agreement between professions. Mental health providers also identified these same categories as ones which lawyers or legal professionals asked them to include in their forensic assessments, only in a slightly different order of frequency: behavioral assessment/demeanor (51.92%); a specific diagnosis of neurocognitive/developmental disorders (26.92%); character assessment (26.92%); and rehabilitation of addiction (23.08%). The inclusion of these components in the mental health forensic report may point to legal professionals’ preoccupation with biases that legal norms or immigration adjudicators bring to assessments of credibility. For example, legal norms in asylum cases consider the migrant’s “demeanor” (without defining the term) as one of the factors that is relevant in the assessment of credibility.^70^ Yet, there is no agreed upon definition of demeanor among adjudicators nor is there a common understanding on what the term means across professions, including as between lawyers and mental health professionals.^71^ As a result, immigration adjudicators draw conflicting conclusions on issues of credibility based on similar expressions of demeanor.^72^ Moreover, science-based studies suggest that relying on expressions or other modes of communication are poor indicators of truth-telling or lying, especially given that cultural differences might explain distinct demeanor practices that could be misread between individuals of different cultures.^73^ Similarly, addictions to alcohol or even past behavioral misconduct, which might be consistent with the impact of trauma in the victim’s life,^74^ could instead be viewed by the immigration adjudicator as indicators of the migrant’s unreliability to tell the truth.^75^

### D. Professional Understanding of Concepts Related to Credibility

The relative newness of the use of forensic immigration assessments in immigration adjudication led us to include questions in the survey that sought to understand how “lawyers and legal professionals” and “mental health practitioners” understood key terms across their respective disciplines. In our survey, we focused on three key terms: (1) credibility and (2) malingering, which we asked about together since they are related but distinct concepts, and (3) the Istanbul Protocol.^76^

Credibility was of special interest to us because, while the legal concept of credibility can be the crux of an immigration claim, the concept does not exist in the terminology of mental health assessments. A sister term to credibility that mental health practitioners use is malingering, but it does not mean the same thing. Malingering is a type of deceptive behavior in psychology that involves intentionally feigning illness or injury or exaggerating symptoms for secondary gain.^77^ The secondary gain might include immigration relief or access to medical or other benefits the client believes they can obtain by exaggerating psychological symptoms or trauma.

In contrast to malingering, credibility is a legal determination about whether a petitioner seeking an immigration benefit or relief is telling the truth about their experiences of trauma.^78^ Only immigration adjudicators are positioned to derive this legal determination, usually through an assessment of factors that include demeanor, plausibility, and consistency of their story of trauma.^79^ An immigrant who is malingering may also be lying about their trauma, but this is not necessarily so. The two concepts are simply not the same. Someone may be truthful about the past or present circumstances of trauma (credible) while at the same time exaggerating their symptoms (malingering). Many factors, other than intentionally lying to gain a benefit, may explain malingering. A client, for example, may have a psychological condition, such as borderline personality disorder, that may lead them to tell their life story in dramatic or erratic ways.^80^ Others may feel compelled to be loud about their symptoms in order to be taken seriously, as may be the case for women of color who are used to being dismissed by medical professionals,^81^ or it may be that cultural or linguistic differences can mean clients lack the language to describe their mental health symptoms or trauma in a way that translates into acceptable western social constructs of psychological issues.^82^ For example, certain cultures tend to experience and/or describe psychological distress in terms of physical ailments, such as stomachaches, headaches, or even heart problems.^83^ None of this, however, is being done to deceitfully gain a benefit, including an immigration benefit, nor is it inconsistent with the presence of past or current trauma. Importantly, mental health practitioners cannot draw conclusions about whether a story their client shares did or did not happen. Instead, mental health practitioners can only assess whether a person’s clinical presentation at the time of the evaluation is consistent with the experiences of trauma they describe.^84^

We first asked “lawyers or legal professionals” and “mental health practitioners” whether they think there is a difference between the terms malingering and credibility. Of the lawyers (N=77), forty-nine (63.64%) replied “yes.” Twenty-six respondents (33.77%) said they were unsure, and only two (2.6%) said “no.” Responses from mental health practitioners (N=46) were more split. Twenty-one respondents (45.65%) were unsure, and another seven (15.22%) said there was no difference between the two terms. Only eighteen respondents (39.13%) confidently said that the terms differed. This demonstrates a potential interprofessional misunderstanding of key terms and concepts necessary to building a sound immigration case and responsibly assessing and communicating the impact of trauma. The open-ended question that followed asking respondents to explain the differences between malingering and credibility revealed a range of different understandings of the terms. Among mental health professionals, the greatest range of divergent answers was in their explanation of credibility, which also implied different understandings of their role in assessing it. Respondent 1, for example, explained credibility as the “consonance between the client’s psychosocial history and the reported and observed psychological symptoms.” In contrast, Respondent 6 stated that credibility is about “consistency and forthrightness.” If these answers are assessed against the Istanbul Protocol, Respondent 6 is ignoring the Protocol’s caution that inconsistencies are likely to arise in accounts of torture or ill-treatment for a host of reasons unrelated to credibility and that the duty of the mental health clinician is to explain or clarify those inconsistencies as part of the assessment.^85^ In contrast, Respondent 1 is more consistent with the Protocol’s recommendation that when asked to assess credibility, a mental health expert should consider the “reliability of the clinical evidence and the extent that it is consistent or inconsistent with allegations of torture or ill-treatment.”^86^

Legal professionals also had a range of divergent responses to how they understood the convergence and divergence of the terms. Many understood that malingering involves the exaggerating of mental health symptoms, and a few said it was a fabrication of facts. Most also understood credibility as the ability to tell a story in a believable way, without necessarily attributing actual truthfulness to the term. The more interesting responses from legal professionals attempted to explain the relationship between malingering and credibility. Respondent 4, for example, viewed malingering as precluding credibility, stating: “I think malingering is a whole other extreme where a person is completely lying about what she or he is suffering. I believe malingering is at the extreme of the credibility spectrum.” As we explained above,^87^ however, this is not necessarily the case. Respondent 11, in contrast, stated: “Malingering, to me, is the exaggeration specifically related to symptomology. Credibility may be harmed, but it is not automatically negated, by malingering.”

In our survey we were also interested in documenting the degree to which respondents, both legal and mental health practitioners, were aware of and applied the Istanbul Protocol in their work. The Istanbul Protocol is increasingly being adopted in asylum and other types of immigration adjudication and, as a multidisciplinary guide that combined the knowledge of scientific experts and practitioners in designing best practices, is considered the gold standard to apply not only to the documentation of torture, but of trauma generally.^88^ During his keynote address at a conference organized by some of the authors of this paper addressing the topic of bridging the scientific-legal divide in the adjudication of trauma,^89^ Dr. Juan E. Méndez, former U.N. Special Rapporteur on Torture and Other Cruel, Inhuman or Degrading Treatment and editor of the Istanbul Protocol, discussed with the audience how important the Istanbul Protocol had become to the adjudication of asylum more broadly.^90^

Responses to our survey questions — both about the relationship between credibility and malingering, and about familiarity with and application of the Istanbul Protocol — reveal surprising gaps in knowledge among both legal and mental health practitioners who are engaging with immigration forensic evaluations. Despite the importance of the Istanbul Protocol in the documentation of trauma, most legal professionals and a significant share of mental health practitioners who responded to our survey had never heard of it. When we asked “lawyers and legal professionals” if they were familiar with the Istanbul Protocol (N=78), fifty-four (69.23%), a large majority, responded “no,” with another four (5.13%) stating they were unsure. Only twenty respondents (25.64%) replied “yes.” The proportions were approximately reversed for mental health practitioners (N=38), with twenty-five (65.79 %) responding “yes,” eleven (28.95%) responding “no,” and another two (5.26%) being unsure.

Mental health practitioners who indicated that they were familiar with the Istanbul Protocol were asked a follow-up question about whether they applied it in their immigration forensic assessments. Sixteen (66.67%) respondents asked this question (N=24) said “yes,” while five (20.83%) indicated that they were unsure whether they applied it in their assessments. Three mental health practitioners (12.5%) said they did not apply the Protocol in their assessments. Given that the Istanbul Protocol is the gold standard — which many leaders in the field assume is being referred to and applied in trauma adjudication — it is telling that, in total, fewer than half of mental health evaluators (42.11%) surveyed indicated that they were both familiar with the Protocol and applied it in their assessments, and just over a quarter (25.62%) of lawyers and other legal professionals surveyed (all of whom were actively practicing immigration law and seeking mental health forensic evaluations) indicated that they confidently knew what it was. These data suggest a need to reinforce the teaching of the Istanbul Protocol to both mental health and legal professionals as part of their degree curricula and likely as part of training while in practice.

### E. Credibility and Case Outcomes

A key focus of our survey was on the relevance of mental health immigration forensic assessments to credibility adjudication in immigration cases; thus, we sought to understand respondents’ own perceptions of how much credibility mattered to immigration outcomes. We asked legal professionals how often their clients were denied relief based wholly or in part on adverse credibility determinations. This is a question we only asked of legal professionals, since in most cases, mental health evaluators are not aware of the ultimate outcome in cases in which they provided assessments. Most legal professionals (N=75) indicated that adverse credibility determinations affected outcomes at least sometimes (56%), while another 6.67% said about half the time, and 5.33% said most of the time. About a third of respondents (32%) replied that adverse credibility determinations never (in whole or in part) explained the denial of relief for their clients. Thus, at least two-thirds of the respondents view adverse credibility determinations as relevant to their clients’ case outcomes.

There are times legal professionals may receive feedback from adjudicators during a negative finding. Therefore, we asked legal professionals what evidence or reasons immigration adjudicators cite when making adverse credibility findings, and, moreover, whether legal professionals had concerns about the indicators decision-makers use to evaluate credibility. Consistent with the Real ID Act^92^ — which codifies the legal standard for assessing credibility in asylum cases^93^ — we offered legal professionals five choices, along with an “other” category, to indicate the reasons adjudicators cite for adverse credibility determinations. Respondents were allowed to select multiple choices. The first four choices are part of the Real ID Act, while we added a fifth category to gauge the relevance of immigration forensic assessments in the decision-making process. Those categories were as follows: (1) inconsistency with client story/stories; (2) inconsistency between client story and evidence; (3) client demeanor; (4) lapses in client memory; and (5) inconsistency between client story and forensic evaluation. Sixty-six respondents provided a total of 202 responses noting the following: (1) inconsistency within client story/stories — 89.39%; (2) inconsistency between client story and evidence — 77.27%; (3) client demeanor — 53.03%; (4) lapses in memory — 46.97%; (5) inconsistency between client story and forensic evaluation — 19.70%; and other — 19.70%. Respondents to the “other” category generally indicated issues with plausibility or lack of experience of a rejected case.

We do not know from these responses whether the adverse credibility determination occurred in cases that included a forensic immigration evaluation or not; and thus, whether including these reports could have helped change the outcome.^94^ It is interesting to note, however, that it is possible that the inclusion of a mental health immigration report can yield its own inconsistencies that could, theoretically, adversely impact the credibility determination as evidenced by the thirteen respondents who indicated that had experienced inconsistency between the immigrant’s story and a forensic evaluation leading to an adverse determination.

### F. Perceptions by Profession of Credibility Indicators

As indicated above, the vast majority of legal professionals — two-thirds of the respondents — view adverse credibility determinations as relevant to their clients’ case outcome.^95^ Unsurprisingly, an even higher proportion of legal professionals surveyed (75% of N=60 respondents) reported having reservations or concerns about the indicators that immigration adjudicators use to determine credibility.^96^ The open-ended question that followed asking respondents to explain their reservations or concerns about the indicators highlighted cultural nuances, differences in trauma presentation with the applicant, neurocognitive deficits, psychiatric presentation (e.g., schizophrenia), and the legal standard allowing for minor inconsistencies to be utilized as a reason for denial by the adjudicator. Respondent 2 noted, “Judges frequently deny based on very small differences in testimony and other interviews/documents, or on ’plausibility,’ which is impossible to refute.” Respondent 37 stated, “I don’t think that demeanor should be a factor. I have plenty of traumatized clients with flat affect, or whose outward emotions don’t seem in line with what they’ve experienced. I also don’t like that the judges are so concerned with details like time or order of events, given that traumatized folks struggle with recalling that type of information.” Lastly, legal professionals expressed concern over the emphasis on consistency. As suggested by respondent 23, “Consistency is highly overrated, particularly when it relates to details that don’t go to the ‘heart of the claim.’”^97^

Thus, in our survey we explored respondents’ perceptions of which factors are relevant to credibility and how their own understanding diverges or converges with the factors relied upon by other professions and/or adjudicators. In each of the respective surveys, we first asked legal and mental health professionals what indicators *they* viewed as relevant to the determination of credibility. We further asked them what indicators they perceived that adjudicators view as relevant for determination. We provided thirteen choices that combined both relevant legal norms and mental health evaluation norms or practices and provided the same to both sets of survey respondents, irrespective of professional affiliation. By design, we did not adapt the indicators we provided to the different disciplines, nor did we provide working definitions for any of the choices or expect that all choices were relevant to their discipline and training.

The parallel questions in the surveys were intended to capture the interdisciplinary differences that may explain different understandings of, and approaches to, credibility assessments. The choices were: emotional/behavioral congruence with personal story; consistency with psychological/neurocognitive models of trauma; consistency with testimony by family or other witnesses; factual consistency in personal story; temporal consistency in sequence of events; validity scales; effort testing; sufficiency of detail and specificity; plausibility; consistency across interviews/declarations/etc.; demeanor; consistency with corroborating evidence; and availability of collateral/corroborating evidence. For each of these choices, we asked respondents to indicate whether they considered these: extremely important; very important; moderately important; slightly important; not at all important; or to select “not applicable/I don’t know what this is.” To allow direct comparison across groups, we assigned a numeric value to each of the ratings, from 1 (not at all important) to 5 (extremely important). Responses of not applicable/I don’t know were removed from these calculations.

Survey responses indicate there is overlap, but with important variations, in what legal professionals and mental health providers consider key indicators of credibility (Table 3). The top five most important factors as rated by mental health practitioners, in order of the average-rated importance were: (1) consistency with psychological/neurocognitive models of trauma; (2) emotional/behavioral congruence with personal story; (3) consistency with corroborating evidence; (4) consistency across interviews/declarations/etc.; and (5) demeanor. Of these, only consistency with psychological/neurocognitive models of trauma was given an average rating of 4 (very important) or above. Attorneys, on the other hand, rated all five of their top indicators as a 4 or above. Listed in the order of rated importance, these were: (1) consistency with corroborating evidence; (2) consistency across interviews/declarations/etc.; (3) factual consistency in personal story; (4) sufficiency of detail and specificity; and (5) plausibility. It is also important to note that mental health professionals did not rate effort testing (M = 2.5) or validity scales (M = 3.04) as particularly important to assessing credibility, and a large proportion of these professionals (validity scales = 25%; effort testing = 61.11%) in fact chose “N/A - I don’t know what this is” when rating these factors. This is concerning in that it indicates that many professionals conducting these evaluations are either not aware of these standard psychological evaluation tools, or do not find them important. This suggests that at least a fair proportion of the individuals conducting evaluations may not have sufficient training in psychometric evaluation.

**Table 3.** Average rated importance of credibility indicators for mental health evaluators (N=38) and legal professionals (N=77),^98^ presented as mean (sd)^99^

Importance Ratings of Credibility Indicators		
Indicator	Mental Health Evaluators	Legal Professionals
Emotional/Behavioral Congruence with Personal Story	3.92 (1.12)	3.55 (1.06)
Consistency with Psychological/Neurocognitive Models of Trauma*	4.11 (0.92)	3.64 (1.01)
Consistency with Testimony by Family or Other Witnesses	3.57 (0.98)	3.78 (0.95)
Factual Consistency in Personal Story*	3.61 (0.89)	4.08 (0.87)
Temporal Consistency in Sequence of Events*	2.89 (1.15)	3.63 (0.99)
Validity Scales	3.04 (1.09)	3.39 (1.23)
Effort Testing	2.5 (1.22)	3.24 (1.2)
Sufficiency of Detail and Specificity*	3.58 (1.06)	4.04 (0.93)
Plausibility*	3.42 (1)	4 (0.96)
Consistency across Interviews/Declarations/etc.	3.71 (1.01)	4.11 (0.94)
Demeanor	3.63 (1.05)	3.41 (1.04)
Consistency with Corroborating Evidence*	3.76 (0.79)	4.16 (0.75)
Availability of Collateral/Corroborating Evidence*	2.78 (1.18)	3.37 (1.12)

When directly comparing ratings^100^ for each of the thirteen factors, we found that seven differed significantly between groups. These were: consistency with psychological/neurocognitive models of trauma, *t*(81.59) = 2.44, *p_cor_* = .037; factual consistency in personal story, *t*(72.56) = -2.71, *p_cor_* = .037; temporal consistency in sequence of events, *t*(62.83) = -3.35, *p_cor_* = .018; sufficiency of detail and specificity, *t*(66.34) = -2.28, *p_cor_* = .048; plausibility, *t*(66.77) = -2.92, *p_cor_* = .031; consistency with corroborating evidence, *t*(71.74) = -2.57, *p_cor_* = .037; and availability of collateral/corroborating evidence, *t*(67.99) = -2.51, *p_cor_* = .037. As a whole, these findings demonstrate that while legal and mental health professionals agree on the importance of consistency of the petitioner’s story across contexts and with available corroborating evidence, attorneys tend to emphasize more specific aspects (e.g., factual, temporal, degree of detail) of narrative consistency to a higher degree. This highlights the lack of knowledge of legal standards for credibility on the part of mental health practitioners who are more focused on the consistency between the experiences described, the petitioner’s clinical presentation,[Fn fn101] and current scientific understanding of trauma rather than factual importance of the case. What is less clear is whether respondents have considered which indicators *should* matter, and specifically whether the indicators they consider important are consistent with current scientific understanding of how trauma might impact a petitioner’s ability to narrate their own story. On the other hand, the indicators endorsed by legal professionals clearly reflect the current legal standard of a credible story: one that is consistent, complete, and linear, but do not reflect current scientific understanding of how trauma might impact a petitioner’s ability to narrate their own story. Furthermore, a lack of interprofessional agreement on the most important indicators of credibility may lead to challenges in coordinating the various aspects of a case and cause frustration or communication failures between attorneys and evaluators (particularly if each does not understand the other’s background and perspective), which could ultimately have a negative impact on the applicant’s chances of a successful petition. Whether these interprofessional differences stem from a lack of trauma-informed education for legal professionals, or from attorneys’ greater awareness of the legal standards, a takeaway is that clear communication and cross-education between professions is warranted.

To assess whether respondents were aware of these interprofessional differences in perceptions of credibility, we asked both legal and mental health professionals if they thought the indicators they use to evaluate the credibility of an immigrant’s story differed from those relied on by the other group, or by immigration adjudicators. Unfortunately, we asked this as a composite question and did not seek clarification as to whether the perceived differences were between themselves and legal or mental health professionals, respectively, or between themselves and adjudicators. Regardless, the answers reveal that most respondents on both surveys are aware of these interprofessional differences: 66.67% of mental health professionals (N=36) and 73.21% of legal professionals (N=72) answered “yes.” Another 30.56% of mental health and 16.67% of legal professionals responded that they were unsure. Only 2.78% of mental health and 9.72% of legal professionals said “no.”

We then asked mental health and legal professionals which indicators of credibility, in their experience, adjudicators consider important (Table 4). The top five indicators that mental health professionals say adjudicators think are important are: (1) consistency across interviews/declarations/etc.; (2) plausibility; (3) factual consistency in personal story; (4) consistency with corroborating evidence; and (5) temporal consistency in sequence of events. Of note, the only three factors that mental health evaluators said were below a “very important” to adjudicators were consistency with psychological/neurocognitive models of trauma — the factor that mental health professionals themselves rated as most important — and the two psychometric testing-specific items: validity scales and effort testing. This is a striking divergence — the fact that evaluators rated the factor that they thought was most important as the *least* important to adjudicators, followed by the two items specific to their profession suggests that they are aware of the divergence between their own professional perspective and that of adjudicators. It also may indicate that they do not experience adjudicators as trauma-informed or aware of the standards of psychological evaluation. It is possible that this conveys a degree of frustration with the elements of credibility adjudicators focus on. Supporting the possibility of uncertainty about adjudicator priorities on the part of evaluators, of the thirty-eight mental health professionals who responded to this question, twelve to fifteen consistently responded to each indicator with “N/A - cannot assess or do not know” (with a whopping twenty-five responding N/A to effort testing and seventeen to validity scales) suggesting that around thirty to forty percent of our mental health respondents do not have a clear sense of what indicators adjudicators find important in making credibility determinations.

**Table 4.** Average perceived importance to adjudicators of credibility indicators, presented as Mean (sd)^101^

Perceived Importance to Adjudicators of Credibility Indicators		
Indicator	Mental Health Evaluators	Legal Professionals
Emotional/Behavioral Congruence with Personal Story	4 (0.78)	4.19 (0.86)
Consistency with Psychological/Neurocognitive Models of Trauma	2.96 (1.17)	3.2 (1.38)
Consistency with Testimony by Family or Other Witnesses	4.17 (0.65)	4.45 (0.64)
Factual Consistency in Personal Story	4.72 (0.46)	4.79 (0.44)
Temporal Consistency in Sequence of Events	4.44 (0.77)	4.58 (0.58)
Validity Scales	3.35 (1.39)	2.96 (1.43)
Effort Testing	3 (1.54)	2.7 (1.34)
Sufficiency of Detail and Specificity	4.42 (0.7)	4.6 (0.63)
Plausibility	4.72 (0.46)	4.66 (0.53)
Consistency across Inerviews/Declarations/etc.	4.88 (0.33)	4.84 (0.4)
Demeanor	4.21 (0.72)	4.16 (0.85)
Consistency with Corroborating Evidence	4.54 (0.58)	4.68 (0.58)
Availability of Collateral/Corroborating Evidence	4.33 (0.92)	4.13 (0.91)

**Table 5.** The degree to which mental health evaluators (N=37) report adjusting their approach to assessing credibility based on different factors, alongside how much mental health evaluators (N=38) and legal professionals (N=77) perceive that adjudicators adjust their approach based on different factors.^109^ Reported as mean (sd)

How Much Are Different Factors Taken Into Account When Assessing Credibility?			
Factor	Mental Health Evaluators	Evaluators’ Perception of Adjudicator	Legal Professionals’ Perception of Adjudicators
Cultural Factors	4.16 (0.69)	2.25 (0.61)	2.21 (0.82)
Types of Harm/Trauma Experienced	4.14 (0.79)	2.72 (0.84)	3.03 (1.05)
Gender-related Factors	4.11 (0.74)	2.33 (0.92)	2.57 (0.98)
Religious Factors	3.73 (0.96)	2.5 (0.98)	2.51 (0.97)
Linguistic Factors	4.05 (0.97)	2.32 (0.75)	2.32 (0.84)
Medical Factors	3.74 (0.92)	3.19 (1.17)	3.12 (1.1)
Disability Status	3.73 (1.04)	3 (1.09)	2.88 (1.13)
Prior Criminal History/Interactions with Criminal Justice			
System	3.25 (1.19)	4.16 (1.14)	3.99 (1.08)
Other	4.4 (0.55)	2.5 (0.71)	4 (NA)

Legal professionals’ responses showed a similar pattern of perceived adjudicator emphasis (Table 3). The top five indicators by average perceived importance rating were: (1) consistency across interviews/declarations/etc.; (2) factual consistency in personal story; (3) consistency with corroborating evidence; (4) plausibility; and (5) sufficiency of detail and specificity. In another point of agreement with mental health evaluators’ perceptions, legal professionals indicated that, in their perception, only three indicators were less than “very important” to adjudicators. These were, once again, the indicators specific to mental health and psychometric evaluation: consistency with psychological/neurocognitive models of trauma, validity scales, and effort testing. A smaller proportion of legal professionals indicated uncertainty about adjudicator priorities, with three to eight respondents consistently choosing “N/A - cannot assess or do not know” for the various indicators, suggesting that approximately four to eleven percent of legal professional respondents do not have a clear sense of adjudicator priorities. As with the mental health professionals, many respondents chose “N/A” for effort testing (55) and validity scales (51), though this likely reflects, at least in part, their own lack of knowledge about what these are rather than their perceptions of adjudicators.

This high degree of agreement between mental health evaluators and legal professionals about which indicators adjudicators emphasize is confirmed by direct statistical comparisons: none of the perceived importance ratings were significantly different between groups. These findings demonstrate that, in general, the great majority of legal and mental health professionals who feel qualified to assess adjudicator priorities agree that immigration adjudicators attach great importance to factors that relate to consistency as well as plausibility, emphasizing facts and how these are presented in the client narrative. Moreover, the large proportion of mental health professionals, in contrast to lawyers, who consistently indicated that they are not able to assess how important adjudicators think different credibility indicators are suggests that this may be a priority area for interprofessional educational efforts.

Conversely, when comparing the importance that evaluators and legal professionals report placing on the different indicators themselves versus the importance they perceive adjudicators placing on different indicators, both groups’ responses significantly differ for nearly all indicators.^102^ In fact, the only indicators that do *not* show statistically significant differences between each groups’ own importance ratings and their perceptions of adjudicators’ emphasis are validity scales and effort testing for both groups, and emotional/behavioral congruence with the personal story for mental health evaluators only (all corrected *ps* > .05). Overall, these findings suggest that both mental health and legal professionals experience their perceptions of what is important to determining credibility as different from what adjudicators find important.

### G. Evaluating Credibility

Through the survey, we also sought to understand how mental health professionals assess or address credibility as part of their immigration forensic assessments. The Istanbul Protocol does not recommend that mental health professionals comment directly on issues of credibility; rather the Istanbul Protocol states that:

Clinical opinions on the credibility of an alleged victim or suspect should be considered in light of the clinician’s expertise and circumscribed, if possible, to the reliability of the clinical evidence and the extent to which the clinical evidence is consistent or inconsistent with specific allegations of torture or ill-treatment … . In situations in which courts request or require a clinician to render an opinion on the credibility of individuals, rather than the clinical findings, the clinician should note that the credibility assessment of an individual is beyond the scope of the Istanbul Protocol, which advises that clinical opinions should be limited to opinions on the reliability of the clinical evidence and the extent to which the clinical evidence is consistent or inconsistent with specific allegations of torture or ill-treatment.^103^

The Istanbul Protocol further states that “[i]f the clinician is asked by a legal expert to provide an assessment of credibility, the clinician should provide their assessment of the reliability of clinical evidence as it relates to credibility and be sure to distinguish their assessment and opinion from a judicial determination of credibility.”^104^ The Istanbul Protocol further specifies that a mental health evaluation (psychological forensic report) should contain a “clinical opinion on the possibility of torture or ill-treatment based on all relevant clinical evidence, including physical, psychological findings, historical information, knowledge of regional practices of torture, consultation reports, etc.”^105^ Indeed, the overarching purpose of the medico-legal evaluation, according to the Istanbul Protocol is:

[T]o provide a clinical interpretation of the degree to which clinical findings correlate with the alleged victim’s contention of abuse, and a clinical opinion on the veracity of such claims, and the possibility of torture, based on all relevant clinical evidence, and to effectively communicate these findings, interpretations and conclusions to the judiciary or other appropriate authorities. In addition, clinical testimony often serves to educate the judiciary, other government officials and the local and international communities about the physical and psychological sequelae of torture.^106^

However, we knew from the mental health forensic experts who formed part of our team that U.S. legal professionals do ask mental health professionals to assess credibility as part of their report. We confirmed this in our study. In the survey for legal professionals, we asked them if mental health evaluators include an explanation of how they approach assessing client credibility as part of their reports. The majority of legal professionals (N=77) answered in the affirmative. Only 16.88% replied either no or that they were unsure whether mental health immigration forensic reports discuss issues of credibility assessments. Similarly, we asked mental health evaluators (N=39) whether they explain the approach they used to evaluate credibility as part of their written reports. Only 5.13% of respondents said they never provided explanations; among the rest, 35.90% said always or most of the time, while 23.08% said sometimes. The most common answers in the open-ended question asking about the content of the explanation described the methodology employed to conduct the assessment and whether the client’s mental health condition and presentation (demeanor) correlates to the presence of trauma in their lives. This could indicate that clinicians believe they are addressing questions of credibility when they are instead discussing the reliability of their opinion and consistency in their findings.

For the remainder of the survey instrument pertaining to how credibility is assessed, we asked questions of only mental health providers. The first question we posed to mental health providers was simply how they evaluate questions of credibility when requested by attorneys. For this question, we provided four choices, and asked respondents (N=45) to choose all that applied: (1) behavioral observations; (2) corroborating/collateral evidence; (3) psychological testing; and (4) other. The overwhelming majority of respondents (93.33%) selected “behavioral observation,” and another 80% chose “corroborating/collateral evidence.” The other two choices were less common. Only 44.44% of respondents indicated that they conduct “psychological testing,” and another 24.44% chose “other.” Open-ended responses to the “other” category varied and included factors like paying attention to specificity or contradictions, while acknowledging that trauma affects memory. This highlights a disconnect between attorneys’ lack of understanding of mental health clinician’s ethics and boundaries, as mental health clinicians lack an understanding of the legal standard of credibility. Respondent 3, consistent with the Istanbul Protocol, is the only one who categorically stated that their role is not to evaluate credibility: “As the expert, I never offer opinions about credibility. Rather I offer opinions about symptoms/functional impairments, which I think should inform credibility determination, but leave credibility determination to whoever reads my report.”

We then asked a few additional questions flowing from the first framing question. We wanted to know what types of corroborating/collateral evidence mental health providers tended to use to assess credibility. Respondents who indicated that they use corroborating/collateral evidence were provided six specific types of evidence and asked which they use: (1) declarations; (2) family interviews; (3) medical records; (4) legal and law enforcement records; (5) educational records; and (6) other. Respondents (N=36) were allowed to choose as many of the relevant choices as they wanted. The majority of respondents chose “declarations” (80.56%); “family interviews” (75%); “medical records” (69.44%); and “legal and law enforcement records” (58.33%). Another 38.89% chose “educational records,” while 25% chose “other.” Mental health clinicians appear to be relying on a wide range of collateral information to inform their clinical opinions.

We then asked respondents who indicated that they employ “psychological testing” (N=20) to evaluate credibility to select what types they use from a menu of nine choices: (1) symptom checklist; (2) trauma assessments; (3) malingering scales (standalone); (4) personality assessments; (5) cognitive/intellectual assessments; (6) effort testing (standalone); (7) embedded effort testing; (8) projective measures; and (9) other. Here too, respondents could choose as many of the relevant choices as they wished. We learned that the majority of mental health providers who engage in psychological testing are largely conducting two types of psychological testing: “symptom checklists” (80%) and “trauma assessments” (75%). Another forty percent chose “malingering scales,” and thirty-five percent stated “conducting personality assessments.” Twenty-five percent noted “cognitive/intellectual assessments,” another fifteen percent noted “effort testing (standalone),” and another ten percent each selected “embedded effort testing” and “projective measures.” Finally, twenty-five percent also selected “other.” Together, these findings tell us that less than half (~44%) of mental health evaluators who responded to our survey use psychological testing, and the majority of those who do appear to rely on symptom checklists, which does not take into account the potential for over or under exaggeration or feigning of symptoms that could account for inconsistency in credibility.

We also asked mental health professionals who indicated that they use behavioral observations to assess credibility (N=42) about the specific types of observations they use, asking them to select all that apply from a menu of ten choices that included: (1) eye contact; (2) mental status exams; (3) speech; (4) evasiveness; (5) nervousness; (6) question style response; (7) defensiveness; (8) motor movement; (9) physiological testing; and (10) other. Many of these behavioral items would be considered elements of “demeanor” in the immigration context, which is considered relevant by immigration adjudicators when assessing credibility. As such, of the areas we directly asked about in our survey, this question comes closest to approximating what immigration adjudicators label “demeanor “in their own assessments. Demeanor is not well-defined in law, and analysis shows that decision-makers draw contradictory conclusions in cases based on observations of similar types of demeanors.^107^ We sought to understand whether this practice of relying on demeanor is also present in mental health providers. The majority of choices were endorsed by more than half of respondents: (1) eye contact (71.43%); (2) mental status exams (69.05%); (3) speech (69.05%); (4) evasiveness (64.29%); (5) nervousness (61.90%); (6) question style response (61.90%); (7) defensiveness (59.52%); and (8) motor movement (57.14%). The only exceptions to this were “physiological testing,” chosen by only 11.90%, and “other”, selected by 19.05% as one of their choices. Thus, our data reveals that mental health providers do rely on demeanor to assess credibility, in addition to the other factors they consider. Further research is needed to understand whether mental health providers are using these behavioral observations in consistent ways with one another, as well as how their use compares to how immigration adjudicators rely on demeanor to assess credibility. Clinicians are trained to use behavioral observations (i.e., demeanor) to determine the clinical presentation and rule out diagnostic etiology while adjudicators may utilize demeanor to make a determination on credibility. It is important to note, however, that scientific literature has questioned whether these kinds of behavioral observations should be used at all to assess credibility.^108^ Additionally, mental health clinicians employ their expertise to focus on small nuances and details in behavior, which legal professionals and adjudicators may overlook or not identify. In this sense, legal professionals and adjudicators are not trained on how to interpret demeanor.

### H. Evaluating Credibility Across Sociocultural Difference

A substantial concern of credibility assessments or adjudication, whether by mental health professionals or legal actors, is how differences in human experiences — which may explain how persons present themselves or respond to trauma differently from others — are ignored in ways that adversely impact credibility determinations.^110^ Studies show that sociocultural differences can impact perceptions of credibility.^111^ The Istanbul Protocol affirms this research^112^ and also includes sections that pertain to the unique circumstances of how torture and ill-treatment also affect children and LGBTQ+ persons.^113^ Thus, we were interested in learning whether mental health providers adjust their approach to evaluating credibility based on sociocultural factors. In the survey, we presented mental health professionals with a series of sociocultural factors that influence trauma responses; and thus, should inform whether mental health providers should adjust how they approach assessments of credibility related to trauma. These factors included, in addition to “other”: cultural factors; types of harms or trauma experienced; gender-related factors; religious factors; linguistic factors; medical factors; disability status; and prior criminal history/interactions with criminal justice system.^114^ We allowed each survey respondent to attribute their own meaning to these terms, and admittedly some of these terms may have been interpreted differently. We provided participants with five options and asked them to choose one: completely; a lot; a moderate amount; a little; not at all; and not applicable. To efficiently summarize the data, we once again coded these responses numerically from 1 (not at all) to 5 (completely), removing “not applicable” responses.

In general, mental health professionals reported adjusting considerably in how they approached credibility based on these client differences. Of the factors provided, evaluators ranked cultural factors; types of harm/trauma experienced; gender-related factors; and linguistic factors as all above a 4 (“a lot”) for how much they adjusted their approach. Additionally, those who indicated they also adjusted based on other factors ranked “other” as a 4.4 on average, though this is difficult to interpret as this category was a catchall for anything we did not list, and not all participants ranked or selected “other” in their responses. All other factors were rated between a 3 (“a moderate amount”) and a 4. These results indicate that, overall, mental health professionals are tailoring their evaluation approaches to the needs of individual petitioners. We view this as positive.

Our survey shows that mental health professionals respond to sociocultural factors and shift their approaches to evaluating credibility. This does not mean, however, that immigration adjudicators do as well. As we did not survey immigration adjudicators, we could not ask this question directly; however, we did ask both legal and mental health professionals to offer us their impressions of whether they think immigration adjudicators shift their approach to assessing credibility in response to sociocultural differences. We presented legal and mental health professionals with the same list of sociocultural factors and the same rating scale from “not at all” to “completely,” and once again coded these from 1 to 5, removing “not applicable” responses as described above.

The differences between how much evaluators report adjusting their approach, for which factors, and how much they perceive adjudicators adjusting their approach are — as with reports of indicators of credibility — striking. The only factor for which evaluators perceive adjudicators as adjusting their approach “a lot” or more (rated as a 4 or greater, on average) is “prior criminal history/interactions with criminal justice.” This is the factor that they, themselves, reported adjusting their approach for the least. The only other factors rated above a 3 (“a moderate amount”) on average were “medical factors” and “disability status.” The factor they report the least perceived adjustment for was “cultural factors” — the factor that evaluators themselves report adjusting the most for. Overall, evaluators’ perception of what, and how much, adjudicators alter their approach represents a near-perfect inversion from the factors that evaluators themselves adjust the most.

In general, legal professionals agree with mental health professionals’ perceptions. The factor they said they perceive adjudicators adjusting their approach the most for (3.99 average) was “prior criminal history/interactions with criminal justice,” and the factor they said they adjusted for the least was “cultural factors.”^115^ Thus, both mental health and legal professionals perceive criminal history as being the primary, and most important, factor for which adjudicators adjust their approach to evaluating credibility. While the question did not specify how it matters — influencing the decision negatively or positively — we think that it is likely to reflect a negative bias against credibility, especially given the negative intersections between crime and immigration law.^116^

As with our questions about the importance of various credibility indicators, a large proportion of mental health respondents consistently indicated that they did not know how much adjudicators took different factors into account. For each of the factors, a total of twelve to fifteen of the thirty-eight mental health evaluators responded “N/A - cannot assess or do not know,” indicating that around thirty to forty percent of our mental health respondents do not have a clear sense of if or how adjudicators take these key factors into account in their credibility determinations. This proportion was much lower for our legal professionals: for most indicators only two to three respondents (with a maximum of six on “religious factors”) chose “N/A - cannot assess or do not know,” indicating that only around two to four percent of legal professionals in our sample felt a high degree of uncertainty about how adjudicators adjust their approach to assessing credibility. Given the gap between mental health and legal professionals, training on adjudicator perspectives, priorities, and approaches may represent a ripe opportunity for interprofessional education.

**Plot 1.**
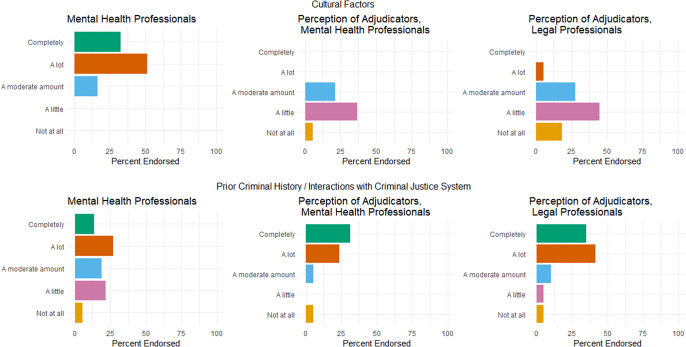
Comparisons of how much different groups adjust their approach to evaluating credibility based on cultural factors and prior criminal history.

### I. Mental Health Diagnosis

A mental health diagnosis is likely helpful to immigrants seeking immigration relief in several respects; importantly, it seems to matter to immigration adjudicators when assessing the credibility of testimony of past or present trauma.^117^ The Istanbul Protocol recognizes the role of a mental health diagnosis in its standards for conducting psychological assessments and recommends that these be used as a way to measure consistency between the psychological findings and the alleged torture and ill-treatment.^118^ However, it cautions evaluators to be mindful of Western biases in medicalizing trauma, instead encouraging evaluators to become familiar with a range of common trauma responses and to expect a diverse range of responses to torture or ill-treatment related to their unique life experiences, their individual coping mechanisms, and the particular cultural, social and political context in which they live.^119^ From our collective experience, based on either our actual completion of immigration forensic assessments or discussions with lawyers who seek them, we anticipated that seeking a mental health diagnosis as part of a mental health immigration forensic assessment would likely be a common practice. Our findings in the survey proved that we were correct in our hypothesis.

We set out to document the practice by asking legal professionals how frequently they explicitly ask for a mental health diagnosis as part of a mental health assessment. Unsurprisingly, the majority of legal professionals (N=77) do so most of the time (38.96%) or always (32.47%). Nearly another quarter (23.88%) said they do so sometimes, with a few (2.60%) doing it about half the time. In fact, only about three percent of legal professionals (2.60%) never ask for a mental health diagnosis. These numbers mirror quite well the responses from mental health professionals (N=36) when asked how frequently legal professionals ask them to include a mental health diagnosis: 38.89% said always; 36.11% said most of the time; 11.11% each said about half the time or sometimes; and only 2.78% said never. Thus, the request to include a mental health diagnosis as part of a mental health immigration forensic evaluations seems to be a common practice. We followed this question up by asking an open-ended question about which mental health diagnoses each group most frequently requests or has requested. Unfortunately, only one legal professional and one mental health professional provided us this more detailed response, so our ability to generalize is extremely limited. Nevertheless, both of these professionals cited PTSD, depression, and anxiety.

Given our hypothesis that legal professionals would frequently seek a mental health diagnosis as part of mental health evaluations (which was confirmed), we also wanted to understand why. Thus, we asked legal and mental health professionals what role they think mental health diagnoses play in mental health immigration forensic evaluations. We provided both groups the same seven choices and asked respondents to pick all that applied: (1) validation of past harm/cruelty/hardship claims; (2) prediction of future behavior/needs/hardship; (3) to explain behavior; (4) assessment of credibility; (5) to explain missed filing dates, failure to appear, or other; (6) potential of rehabilitation; and (7) other. There was substantial overlap in the responses provided by both legal and mental health professionals. Most relevant to the issue of credibility is that over sixty percent of legal (52 out of N=78 respondents) and mental health (23 out of N=37 respondents) professionals alike said mental health diagnoses were important to establishing credibility. An even higher proportion of both groups of respondents (89.74% of legal professionals and 86.49% of mental health professionals) said a mental health diagnosis is relevant to validating past trauma, cruelty or hardship, while another 73.08% of lawyers and 78.38% of mental health providers said they were relevant to explaining behavior.^120^ We certainly do not disagree with legal professionals and mental health providers that a mental health diagnosis is both relevant and useful to establish credibility and, relatedly, strengthen claims of past trauma. However, we worry that the absence of said diagnosis could erroneously become grounds for undermining credibility and claims of past trauma, since, as we have explained, not all victims of trauma necessarily develop a mental health illness as a result.^121^ We did not ask in these surveys whether mental health evaluators take the time to explain why the absence of a mental health diagnosis should not be read to diminish credibility or claims of past trauma, nor did we ask whether their absence yields greater denials of the immigration relief sought. These are important research questions that should be explored further.

### J. Understanding Trauma

Finally, our survey was interested in documenting perceptions across professionals of their own or the other actor’s knowledge of current research about trauma, which we view as important to more accurate assessments of credibility of claims of past trauma.^122^ We first asked legal professionals (N=78) to evaluate their own knowledge of trauma: the majority (51.28%) said they are only moderately knowledgeable, with only 11.54% saying they were very knowledgeable and only 1.28% saying they were extremely knowledgeable. The second largest response of 26.92% of respondents claimed to be slightly knowledgeable, with another 8.97% saying not knowledgeable at all. Thus, over a third of legal professionals who engage with mental health assessments would acknowledge they are lacking knowledge about the science of trauma. Perhaps unsurprisingly, legal professionals’ self-assessment about their own knowledge was more optimistic than mental health professionals’ (N=37) evaluations of legal professionals’ understanding of the science of trauma: only 8.11% found them very knowledgeable, another 29.73% said they were moderately knowledgeable, with none saying they were extremely knowledgeable. The largest proportion (37.84%) said they were only slightly knowledgeable, and another 13.51% said they were not knowledgeable at all. Finally, another 10.81% felt they had no way of evaluating this factor. Quite surprisingly to us, mental health professionals (N=37) evaluated themselves in ways that suggest they also acknowledge their own gaps in understanding the science of trauma, although not to the same extent as legal professionals: 21.62% and 5.41% said they were only moderately or slightly knowledgeable, respectively. Nearly half (48.65%) consider themselves very knowledgeable and another 24.32% said they are extremely knowledgeable.

We next asked about legal and mental health professional’s perceptions of immigration adjudicator’s knowledge of trauma. Legal professionals (N=78) were not exactly optimistic: over forty percent (41.03%) perceive that immigration adjudicators are not knowledgeable at all, while another large portion (39.74%) say they are only slightly knowledgeable. In fact, only 12.82% view immigration adjudicators as moderately knowledgeable to research related to trauma, with none saying they are very or extremely knowledgeable. Another 6.41% did not feel confident in evaluating this factor. Mental health providers (N=37) were equally skeptical that immigration adjudicators possess much knowledge about trauma. An equal 32.43% said they had no knowledge at all or are only slightly knowledgeable (64.86% cumulatively), with another 8.11% saying they are moderately knowledgeable. Only 2.70% considered them very knowledgeable, and none considered them extremely knowledgeable. A quarter of evaluators (24.32%) did not feel equipped to assess this factor. We think it is important for future studies to trace immigration adjudicators’ actual or perceived knowledge of the science of trauma given its relevance to adjudicating credibility about claims of past trauma.

**Plot 2.**
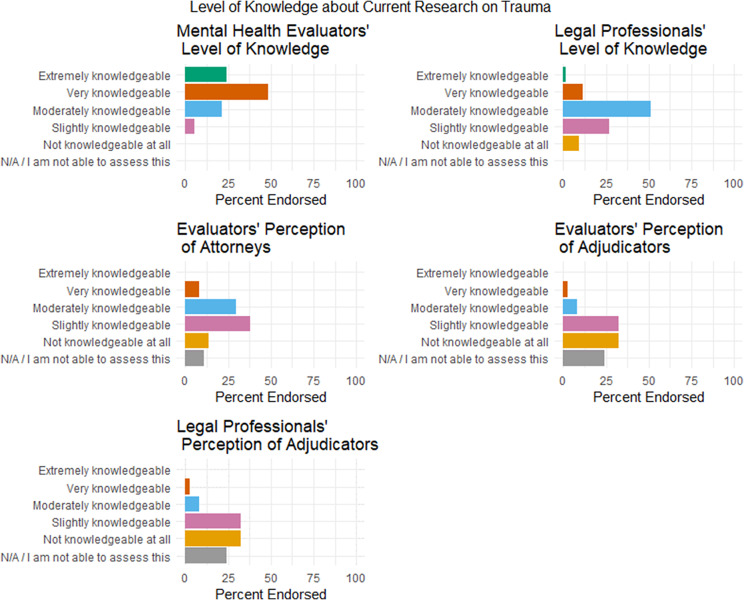
Comparisons of the reported level of knowledge about current research on trauma (particularly regarding memory/reporting).

### A. Key Findings, Limitations and Future Directions

Our research shows that our survey represents the first effort to document how and why mental health immigration forensic assessments are conducted, as well as their role in establishing credibility. The responses we received provided several key insights into the practice of conducting mental health forensic assessments for immigration cases, as well as ways in which the field could improve.

#### Key Findings

The first set of insights relates to access to evaluations and how they are conducted. For the most part, legal professionals felt that including mental health forensic assessments helped their cases, and many would like to include them more often. They cited funding as the most important limiting factor in how often they could seek assessments as most petitioners lack financial resources to pay for assessments themselves. This means that many assessments are sought pro or low bono, which may limit the mental health providers able or willing to offer them.

This leads to the question of who is conducting the assessments and the training they receive. We found that the mental health practitioners conducting forensic immigration assessments come from varied professional backgrounds, with the most common being clinical psychologists (PhD/PsyD) and licensed clinical social workers (LCSW). While many psychological licensure programs contain some training in psychometrics and psychological evaluation, it is not the focus of most specialties. This means that, unless they seek out additional training, professionals may lack expertise about the nuances and limitations of psychological evaluation techniques in general and about how to apply them in immigration and legal settings.

While we did not ask mental health providers about their training or credentials specific to immigration forensic assessments, the responses to other questions indicate that lack of sufficient training may be an issue in our sample. For example, when asked about the importance of validity scales and effort testing — two tools specific to psychological evaluation and psychometrics — to assessing credibility, a considerable proportion of mental health professionals (one-quarter for validity scales and nearly two-thirds for effort testing) indicated that they did not know what these terms meant. This is concerning for a population of practitioners who are actively conducting psychological assessments. Specific to assessment in immigration contexts, when asked about the Istanbul Protocol — which, again, is considered the gold standard for documenting past harm — less than half of mental health professionals surveyed indicated that they applied the Protocol in their evaluations (and just under two-thirds indicated that they even knew what it was). Startlingly, even fewer legal professionals (just over a quarter) indicated that they were familiar with the Istanbul Protocol. This illustrates a significant gap between best practices put forth by experts in the field and what is actually happening on the ground.

The second set of insights relates to the core focus of this paper: interprofessional understandings of credibility and the role that mental health forensic assessments play in establishing credibility in immigration cases. Nearly seventy percent of surveyed legal professionals indicated that adverse credibility determinations affected their case outcomes at least some of the time, demonstrating that it is an important factor to consider in immigration forums. Documenting the impact of trauma was the most frequently endorsed reason for seeking a mental health forensic evaluation for both attorneys and evaluators. Most legal professionals reported that they always or most of the time explicitly request a mental health diagnosis, which they, as well as mental health professionals, viewed as relevant to establishing credibility and validating past harm and trauma. And indeed, the case types reported by the greatest number of mental health and legal professionals tended toward categories — such as asylum and VAWA — that may rest on validating past harm, including psychological trauma. Together, these pieces of information suggest that mental health forensic assessments are one tool advocates may use to try to establish credibility in court.

However, we found inconsistencies across disciplines in terms of what indicators of credibility each group considers most important. Attorneys largely reported privileging credibility indicators having to do with consistency — this includes the facts and flow of events within the story itself, and with external factors such as corroborating evidence. Mental health practitioners were more concerned with consistency with models of trauma and the congruence of behaviors with the petitioner’s personal story. While these different priorities are logical given the focus of different professions, if they play out in different understandings of how to establish credibility, they could create opportunities for miscommunication and hurt case outcomes.

Related to this, there appeared to be lack of clarity around whose responsibility it is to determine credibility. While a trained evaluator is well equipped to assess the consistency of symptoms with the personal history described, credibility is a legal determination made by the court. The Istanbul Protocol outlines this distinction and methods for how evaluators should conduct forensic assessments to contribute to credibility determinations.^123^ However, while mental health respondents reported using a range of methods for evaluating credibility, only one respondent explicitly stated that their role is not to evaluate credibility, but to provide insight into factors that may ultimately be relevant to a credibility determination. This potentially reflects a lack of knowledge about the meaning of credibility in a legal context. As many of our respondents were not familiar with the Protocol, it may be that each group was relying on different, colloquial definitions of credibility in formulating their responses, rather than a shared understanding of the specific meaning of credibility in a legal context. It would be useful for future studies to ask more pointed questions about the meaning of credibility in the context of immigration cases, and whose responsibility it is to assess it to help clarify this issue.

The third and final set of key insights from the current paper relates to perceptions of adjudicators. Seventy-five percent of legal professionals indicated they have some reservations about the indicators that adjudicators use to make credibility determinations. Meanwhile, around thirty to forty percent of mental health practitioners consistently indicated that they were unable to assess how important different credibility indicators are to adjudicators. Among the respondents who did feel they were able to assess which indicators adjudicators find important, there was a high degree of agreement between legal and mental health professionals. In fact, there were no significant statistical differences between the groups in their ratings of relative importance of different credibility indicators to adjudicators. Respondents from both groups indicated that adjudicators care about consistency — whether it be across declarations and interviews, within the story itself, or with corroborating evidence — and plausibility. Both groups indicated that they perceived adjudicators to be less concerned with factors related to psychological testing and neurocognitive models of trauma.

Respondents also shared the overall impression that adjudicators are neither particularly trauma-informed nor sensitive to sociocultural differences in their credibility determinations. While mental health professionals reported adjusting their approach to evaluation based on a range of factors, including sociocultural differences, gender, language, and the type of trauma the petitioner has experienced, neither group of professionals thought that adjudicators made major adjustments to their approach to assessing credibility based on these factors. Rather, the primary factor that both legal and mental health professionals perceived adjudicators as ascribing importance to was the petitioner’s prior criminal history or interactions with the criminal justice system. Similarly, both groups indicated that, in their perception, adjudicators lack knowledge about the science of trauma. Over eighty percent of legal professionals said that they perceived adjudicators as either not knowledgeable at all or only slightly knowledgeable, and over ninety percent of mental health professionals either agreed that adjudicators were not at all or only slightly knowledgeable or said that they were not able to assess adjudicator knowledge. Given that trauma and its effects may be central to many types of immigration cases, this extreme lack of knowledge — whether real or only perceived — on the part of adjudicators is problematic, at best undermining trust in the integrity of the process, and at worst illustrating a significant flaw in our system for determining who qualifies for relief. Further investigating this perceived gap would be a worthy priority for future studies.

#### Limitations

This study has a number of limitations to take into consideration when interpreting our findings. Firstly, this was a relatively small convenience sample of participants, gleaned largely from professional listservs and our own professional networks. This is particularly true of the mental health practitioners, as there is no professional society through which to recruit them. Because of that, our findings may not be generalizable to the whole population of people seeking and conducting mental health forensic assessments.

A second limitation is that we largely asked respondents about their experience *in general*, but not about specific individual cases. That means that while our findings can tell us about the practice of mental health forensic assessments in immigration cases generally, they cannot speak to more nuanced questions about how these practices might vary between cases, for example between different case types or immigration forums.

Finally, a third important limitation is that while we asked about perceptions of adjudicators, we did not survey adjudicators themselves, thus while we have insight into how legal and mental health professionals perceive adjudicators, we do not have direct insight into this group’s own stated priorities and knowledge. Further, when asking about adjudicators, we did not distinguish between judges and administrators. While both adjudicate immigration cases, depending on the forum, there may be important differences between the two that the current survey does not capture.

#### Future Directions

Together, these key findings point to a number of gaps and potential opportunities both for future research and to improve the current state of the field. One obvious next step would be to survey adjudicators directly. Another fruitful area of investigation is cataloguing immigration cases to more effectively map if and how mental health forensic assessments affect case outcomes. This would also allow for better insight into differences between immigration forums. Based on the gaps identified in the current study, developing and disseminating training resources about the science of trauma for immigration attorneys and adjudicators — and about the Istanbul Protocol and credibility determinations for mental health evaluators and attorneys — may present opportunities for immediate improvement within the existing system. It is also clear that there is a need to identify strategies and create structures that allow for more consistent training both within and across disciplines. As they currently appear to be the most dispersed group, the creation of a professional society that could house and disseminate such trainings is one potential starting point. This could also potentially help in identifying existing and garnering new funding sources, which would help to alleviate a major barrier to access. We hope that our initial effort to document current practices can help inform and energize these efforts.

## Conclusion

This study represents a first effort to document the practice of mental health assessments and their role in the adjudication of credibility in immigration cases. The resulting preliminary findings can be useful to guide researchers who may want to go deeper into any of our findings, including, for example, in documenting whether perceptions of immigration adjudicators in these areas are accurate. These findings also suggest the need for more training of all actors in important areas, especially in international norms like the Istanbul Protocol and the science of trauma. This particular finding surprisingly extends to the mental health professionals themselves. We hope these preliminary findings might also help educate both legal and mental health professionals about issues they may not yet have considered and perhaps seek to identify best practices based on collective learning and lessons. Our findings also argue for greater interprofessional communication among legal and mental health professionals who likely have a lot to learn from one another in ways that can improve these types of mental health forensic reports. Ideally, these findings would lead to the identification of best practices and discussion of practices that, while crafted with good intentions, might lead to erroneous practices that are inconsistent with science. Through this survey, for example, we have raised concerns about how important and prevalent mental health diagnoses have become as part of mental health forensic assessments without more critical inquiry as to how these might be creating erroneous expectations among adjudicators about what their presence or absence means regarding trauma. Finally, we hope our preliminary findings can incentivize trauma researchers in particular to identify ways to improve the practice of mental health assessments within the immigration context, and, ideally, help to change norms that are inconsistent with the science of trauma or how the legal field evaluates credibility. Ultimately, we hope that this and future research can lead to legal reforms that are needed to truly improve the adjudication of trauma and assessment of credibility in immigration cases.

## Footnotes

1
*See generally* Raquel E. Aldana et al., *Trauma as Inclusion*, 89 Tenn. L. Rev. 767 (2022).

2
*Id.* at 769.

3
*Id.* at 808, 810. Part II.B of this article reports on the types of immigration cases where forensic immigration reports are procured. Largely, forensic immigration reports are sought to document trauma that has already occurred or that will occur as a result of immigration enforcement, such as family separation. These include immigration cases involving asylum; relief based on instance of crime; domestic violence; child abuse, neglect or abandonment; human trafficking; and family separation. In addition, forensic immigration assessment may be sought to document certain types of learning or other cognitive disabilities, such as in naturalization cases to obtain a waiver from the English language requirement.

4 Raquel E. Aldana, *Repairing Fixable Trauma in Immigration*, *in* Trauma-Informed Law: A Primer for Lawyer Resilience and Healing 86, 86–87 (Helgi Maki et al. eds., Am. Bar Ass’n 2023) (e-book).

5
*See id.* at 87.

6
*Id.*; *see also* Scott Rempell, *Credibility Assessments and the REAL ID Act’s Amendments to Immigration Law*, 44 Tex. Int’l L.J. 185, 218 (2008).

7
*See* Aldana, *supra* note 4, at 87; *see also* Abigail Stepnitz, *Storied Pasts: Credibility and Evolving Norms in Asylum Narratives 1989-2018*, 41 Law & Ineq. 1, 12 (2023) (“Asylum seekers employ available symbols in the practice of narrating their lives to achieve a particular goal: to be recognized as credible and granted refugee status.”); Alana Mosley, *Re-Victimization and the Asylum Process:* Jimenez Ferreira v. Lynch*: Reassessing the Weight Placed on Credible Fear Interviews in Determining Credibility*, 36 Law & Ineq. 315, 321 (2018); Jeanette L. Schroeder, Note, *The Vulnerability of Asylum Adjudications to Subconscious Cultural Biases: Demanding American Narrative Norms*, 97 B.U. L. Rev. 315, 338–40 (2017).

8 David C. Rubin, *Properties of Autobiographical Memories are Reliable and Stable Individual Difference*s, 210 Cognition art. no. 104583, at 10 (2021).

9 Katherine Nelson & Robyn Fivush, *The Development of Autobiographical Memory, Autobiographical Narratives, and Autobiographical Consciousness*, 123 Psych. Reps. 71, 76 (2019).

10 Amy F. T. Arnsten et al., *The Effects of Stress Exposure on Prefrontal Cortex: Translating Basic Research into Successful Treatments for Post-Traumatic Stress Disorder*, 1 Neurobio. Stress 89, 91 (2015).

11 Sabine Schönfeld & Anke Ehlers, *Posttraumatic Stress Disorder and Autobiographical Memories in Everyday Life*, 5 Clinical Psych. Sci. 325, 326 (2017).

12 U.N. Off. of the High Comm’r for Hum. Rts., Istanbul Protocol: Manual on the Effective Investigation and Documentation of Torture and Other Cruel, Inhuman or Degrading Treatment or Punishment, ¶ 287, U.N. Doc. HR/P/PT/8/Rev. 2, U.N. Sales No. E.22.XIV.4 (2022) [hereinafter Istanbul Protocol].

13
*See* Ann Laquer Estin, *Child Migrants and Child Welfare: Toward a Best Interests Approach*, 17 Wash. U. Glob. Stud. L. Rev. 589, 607–08, 611–12 (2018) (describing the shortcomings of the United States immigration system in meeting the U.N. Committee on the Rights of the Child’s “view that children in the context of international migration should be ‘treated first and foremost as children,’” and giving as examples how children typically go through the asylum system without legal representation and there are currently no guidelines for child-sensitive questioning).

14
*See* Nermeen S. Arastu, *Access to a Doctor, Access to Justice? An Empirical Study on the Impact of Forensic Medical Examinations in Preventing Deportations*, 35 Harv. Hum. Rts. L.J. 47, 90–91 (2002) (discussing study of 2,584 immigration cases between 2008 and 2018, in which it was found that applicants who received forensic medical evaluations from PHR were granted immigration relief 81.6% of the time, as compared to a national asylum grant rate of 42.4%, as presented in Holly G. Atkinson et al., *Impact of Forensic Medical Evaluations on Immigration Relief Grant Rates and Correlates of Outcomes in the United States*, 84 J. Forensic & Legal Med. art. no. 102272 (2021)); Stuart L. Lustig et al., *Asylum Grant Rates Following Medical Evaluations of Maltreatment Among Political Asylum Applicants in the United States*, 10 J. Immigr. Minority Health 7, 10 (2008) (conducting a study of a sample of 746 asylum cases between 2000 and 2004 who received forensic medical evaluations from PHR and finding that asylum was granted 89% of the time, as compared to the national average of 37.5% among asylum seekers who did not receive evaluations from PHR).

15 Hans D. Pzoometersen et al., *Physical Torture*, *in* 4 Encyclopedia of Forensic Sciences 182, 197–98 (Max M. Houck et al. eds., 3d ed. 2023).

16
*Id.* at 183.

17 Istanbul Protocol, *supra* note 12, ¶¶ 491, 493, 495.

18
*See, e.g.*, *Trauma & Stressor Exposure Measures*, Nat’l Ctr. for PTSD, U.S. Dep’t of Veterans Affs., https://www.ptsd.va.gov/professional/assessment/te-measures/index.asp [https://perma.cc/6MNB-DWK2] (last visited Feb. 23, 2025).

19 John P. Wilson, *The Lens of Culture: Theoretical and Conceptual Perspectives in the Assessment of Psychological Trauma and PTSD*, *in* Cross-Cultural Assessment of Psychological Trauma and PTSD 3, 3–4 (John P. Wilson & Catherine So-Kum Tang eds., 2007).

20 Edita Fino, Denis Mema & Paola M. Russo, *War Trauma Exposed Refugees and Posttraumatic Stress Disorder: The Moderating Role of Trait Resilience*, 129 J. Psychosomatic Rsch. art. no. 109905, at 2 (2020).

21 Alyssa R. Roeckner et al., *Neural Contributors to Trauma Resilience: A Review of Longitudinal Neuroimaging Studies*, 11 Translational Psychiatry art. no. 508, at 1 (2021) (“Cohort studies tracing symptom levels from hours to years post-trauma show that many people initially experience high levels of depression, anxiety, and posttraumatic stress disorder (PTSD) symptoms in the first days after a traumatic event, but the majority recover naturally.”) (citations omitted); *see also* Alison Mary et al., *Resilience After Trauma: The Role of Memory Suppression*, 367 Science art. no. eaay8477, at 6 (2020).

22 Mark A. Bellis et al., *Life Course Health Consequences and Associated Annual Costs of Adverse Childhood Experiences Across Europe and North America: A Systematic Review and Meta-Analysis*, 4 Lancet Pub. Health e517, e525 (2019).

23 Kathryn Hampton et al., *The Psychological Effects of Forced Family Separation on Asylum-Seeking Children and Parents at the US-Mexico Border: A Qualitative Analysis of Medico-Legal Documents*, 16 PLOS ONE art. no. e0259576, at 6 (2021).

24
*See* sources cited *supra* note 10.

25 Atkinson et al., *supra* note 14, at 9 (Although the pool of trained clinicians and number of medical school asylum clinics are growing, currently Physicians for Human Rights is the largest referral source for pro bono evaluations in the United States, arranging approximately 700 evaluations per year, a small number compared to the tens of thousands of applications submitted each year.”); Richard Rogers & Kamar Y. Tazi, *Challenges and Opportunities for Forensic Mental Health in Immigration Courts*, 53 J. Am. Acad. Psychiatry L. 2, 2–8 (2025) (discussing the need for the American Academy of Psychiatry and the Law (AAPL) to systematize immigration forensic assessments by creating practice guidelines for the evaluation and treatment of immigrant populations).

26 Istanbul Protocol, *supra* note 12, at x.

27
*See, e.g.*, Arastu, *supra* note 14, at 86; Miriam Y. Neufeld et al., *Forensic Evaluation of Alleged Wrist Restraint/Handcuff Injuries in Survivors of Torture Utilizing the Istanbul Protocol*, 135 Int’l J. Legal Med. 583, 583 (2022); Tobias Kelly et al., *A Comparative Study of the Use of the Istanbul Protocol Amongst Civil Society Organizations in Low-Income Countries*, Torture, No. 3, 2016, at 60, 60, 69.

28 Arastu, *supra* note 14, at 86.

29
*Id.* at 84–85. We are not aware of a study documenting whether the Istanbul Protocol is taught to law students at U.S. law schools.

30 Immigration forensic reports are conducted either by medical doctors or mental health providers. While there are similarities in the ways these are conducted, we decided to focus our survey first on mental health providers to capture the uniqueness of this practice. We hope in the future to conduct a similar survey documenting the practice of medical forensic reports.

31 Two members of our team have conducted hundreds of mental health immigration forensic assessments.

32 We posted the request on LinkedIn, Twitter (now X), and Facebook.

33 We sent a request for the completion of the survey to the listservs immprof@lists-ucla.edu and iclinic@list.msu.edu on March 3, 2021. A complete account of our extensive email outreach and other recruitment actions is on file with the authors.

34 Raquel Aldana & Patrick Marius Koga, *Stakeholder Perspectives Report: November 09, 2018 Focus Group Findings on Migrant Legal-Mental Health Intersectionality* 3 (U.C. Davis Off. of Glob. Affs., part of the project *Transforming Refugee Mental Health: Improving Legal Assessment of Credibility*, 2019), https://papers.ssrn.com/sol3/papers.cfm?abstract_id=3625784.

35 We targeted six states: Arizona, California, Florida, Georgia, New York, and Texas.

36
*See* 8 C.F.R. § 1292.1(a)(4) (2024) (the federal rule for accredited representation); Recognition of Organizations and Accreditation of Non-Attorney Representatives, 81 Fed. Reg. 91346 (Dec. 19, 2016); s*ee also* Am. Bar Ass’n Comm’n on Immigr., *Avoiding the Unauthorized Practice of Immigration Law*, Am. Bar Ass’n (July 13, 2021), https://www.americanbar.org/groups/public_interest/immigration/projects_initiatives/fightnotariofraud/avoiding-the-unauthorized-practice-of-immigration-law/ [https://perma.cc/M95E-SE8G] (listing those authorized to practice immigration law: “attorneys, law students and law graduates under the supervision of a licensed attorney, reputable individuals, accredited representatives, and accredited officials”). Our survey intentionally did not include persons who represent immigrants in immigration proceedings who are out of compliance with the regulatory norms that have emerged to regulate non-lawyers.

37
*See generally* William E. Foote, Jane Goodman-Delahunty & Gerald Young, *Civil Forensic Evaluation in Psychological Injury and Law: Legal, Professional, and Ethical Considerations*, 13 Psych. Inj. & L. 327 (2020).

38
*See generally* Daniel A. Martell, *Forensic Neuropsychology and the Criminal Law*, 16 Law & Hum. Behav. 313 (1992).

39 Raquel E. Aldana et al., *supra* note 1, at 821; *see also* Meilynn Shi, Anne Stey & Leah C. Tatebe, *Recognizing and Breaking the Cycle of Trauma and Violence Among Resettled Refugees*, 7 Current Trauma Reps. 83, 87 (2021) (discussing the importance of establishing trauma-informed interventions for refugees and refugee communities in the context of health care and social care spaces).

40
*See* Istanbul Protocol, *supra* note 12, ¶¶ 303, 308.

41
*Id.* ¶ 303.

42
*See* Serena Patel, *Cultural Competency Training: Preparing Law Students for Practice in Our Multicultural World*, 62 UCLA L. Rev. Disc. 142, 144–46 (2014) (advocating for increased cross-cultural competency training for lawyers in law school); Anu Asnaani & Stefan G. Hofmann, *Collaboration in Culturally Responsive Therapy: Establishing a Strong Therapeutic Alliance Across Cultural Lines*, 68 J. Clinical Psych. 187, 189–91 (2012) (suggesting guidelines to facilitate improvement of therapists’ cross-cultural competency strengthening of therapeutic bonds in cross-cultural therapy settings).

43 Erik Ganesh Iyer Søegaard et al., *Gender Differences in a Wide Range of Trauma Symptoms After Victimization and Accidental Traumas: A Cross-Sectional Study in a Clinical Settin*g, 12 Eur. J. Psychotraumatology art. no. 1975952, at 9 (2021); Rebekah Ingram Estevez, *Working with Trauma in Trans and Non-Binary (TNB) Communities: Brief Review of a Burgeoning Literature Base*, 65 Trauma Psych. News (Nov. 13, 2020), https://traumapsychnews.com/2020/11/working-with-trauma-in-trans-and-non-binary-tnb-communities-brief-review-of-a-burgeoning-literature-base/ [https://perma.cc/97GR-3P2A]; Miranda Olff, *Sex and Gender Differences in Post-Traumatic Stress Disorder: An Update*, 8 Eur. J. Psychotraumatology art. no. 1351204, at 1 (2017).

44
*See, e.g.*, Erwin Schweitzer et al., *Gendered Capital in Psychotherapy: A Thematic Analysis of Patients’ Experiences of the Therapists’ Gender*, Counseling & Psychotherapy Rsch. 1357, 1358, 1363 (2024).

45
*See* Jude Mary Cénat, *Complex Racial Trauma: Evidence, Theory, Assessment, and Treatment*, 18 Persps. on Psych. Sci. 675, 676–77 (2023) (arguing that racial trauma must be treated as a unique form of complex trauma that can only be identified by a “life-course approach”); A. L. Roberts et al., *Race/Ethnic Difference in Exposure to Traumatic Events, Development of Post-Traumatic Stress Disorder, and Treatment-Seeking for Post-Traumatic Stress Disorder in the United States*, 41 Psych. Med. 71, 76 (2011) (finding that lifetime prevalence of PTSD varies significantly by race and ethnicity).

46
*See* Thomas G. McGuire & Jeanne Miranda, *Racial and Ethnic Disparities in Mental Health Care: Evidence and Policy Implications*, 27 Health Affs. (Millwood) 393 (2008).

47
*See* Julie D. Lawton, *Am I My Client? Revisited: The Role of Race in Intra-Race Legal Representation*, 22 Mich. J. Race & L. 13, 24–50 (2016) (analyzing the role of race and the importance of race-consciousness separately in inter- and intra-race legal representation); *see also* Russell G. Pearce, *White Lawyering: Rethinking Race, Lawyer Identity, and Rule of Law*, 73 Fordham L. Rev. 2081, 2082 (2005) (“[F]or white lawyers, as well as lawyers of color, increased ‘“competence [in] dealing with racial matters” and “speak[ing] openly, frankly, and professionally about relations”’ is necessary both to competent client representation and equal justice under law.”) (quoting Clayton P. Alderfer, *A White Man’s Perspective on the Unconscious Processes Within Black-White Relations in the United States*, *in* Human Diversity: Perspectives on People in Context 201, 218 (Edison J. Trickett et al. eds., 1994)).

48 George C. Chen, *Beneath the Surface: Why Diversity and Inclusion Matter for Lawyers of Color, and What Lawyers Can Do to Address Implicit Bias in the Legal Profession*, Fed. Law., June 2015, at 28, 30 (“[L]awyers need to actively engage with lawyers of color and support diversity and inclusion efforts. Increased positive exposure to groups who have been associated with negative attitudes and stereotypes can lead to the reduction of implicit bias against individuals in those groups.”).

49
*See* Elena Byhoff et al., *Mental Health Care Use by Ethnicity and Preferred Language in a National Cohort of Community Health Center Patients*, 75 Psychiatric Servs. 363, 363–64 (2024).

50 María J. Marquine & Daniel Jimenez, *Cultural and Linguistic Proficiency in Mental Health Care: A Crucial Aspect of Professional Competence*, 32 Int’l Psychogeriatrics 1, 2 (2020).

51
*See* Doris F. Chang et al., *Rethinking Interpreter Functions in Mental Health Services*, 72 Psychiatric Servs. 353, 353 (2021) (arguing that “[p]rofessional interpreters are crucial for overcoming communication gaps” but that in order to ensure quality of care, “efforts to enhance the outcomes of interpreter-mediated services should transcend basic communication functions to target cultural and interpersonal factors. Fuller use of interpreters’ capacities to overcome linguistic and relational barriers to care can rely on interpreters’ roles beyond the conduit role (simple linguistic conversion). Interpreters are also clarifiers (independently eliciting clarifying information), cultural brokers (providing information on sociocultural contexts), and advocates/mediators (acting on the patient’s behalf to promote quality of care)”); *see also generally* Muneer I. Ahmad, *Interpreting Communities: Lawyering Across Language Difference*, 54 UCLA L. Rev. 999 (2007) (discussing the vital role of interpreters in poverty lawyering and advocating for more collaboration between lawyers, interpreters and clients).

52 Maya Fennig & Myriam Denov, *Interpreters Working in Mental Health Settings with Refugees: An Interdisciplinary Scoping Review*, 91 Am. J. Orthopsychiatry 50, 53, 55–56 (2021) (reporting in a scoping review of literature on refugee experiences with mental health practitioners that many studies found “refugee clients expected to be misunderstood because of the poor quality of interpretation service,” several studies “reported instances of inadvertent mistakes in interpretation and unprofessional interpreter conduct … because of lack of training,” and some studies discussed factors that “can lead clients to believe that interpreters are biased”); Maxwell Alan Miller et al., *Finding Justice in Translation: American Jurisprudence Affecting Due Process for People with Limited English Proficiency Together with Practical Suggestions*, 14 Harv. Latino L. Rev. 117, 124–29, 142–47 (2011) (discussing cases where interpretation introduced errors at trial or where interpretation conducted by police during interrogations introduced bias against the accused); *see also generally* Morten Heath et al., *Interpreter Services and Effect on Healthcare—A Systematic Review of the Impact of Different Types of Interpreters on Patient Outcome*, 7 J. Migration & Health art. no. 100162 (2023) (analyzing relationship between type of interpreter services used and quality of care).

53 According to the U.S. Department of Homeland Security (DHS), the top nationalities seeking asylum affirmatively between 2020 and 2022 were: Cuba, Venezuela, Haiti, Guatemala, Colombia, Honduras, Afghanistan, El Salvador, Nicaragua, and Mexico. In the same period, the top ten nationalities filing for asylum while in removal were: Venezuela, Honduras, Cuba, Guatemala, Ecuador, Brazil, El Salvador, Nicaragua, Mexico, and Haiti. Irene Gibson, Off. of Homeland Sec. Stat., Annual Flow Report: Refugees and Asylees: 2022, 9 tbls.6a & 6b (2022).

54 The other sectors listed by legal professionals were public defender’s offices, solo pro bono volunteer, medical schools, and social service nonprofits.

55 See Aldana et al., *supra* note 1, at 810–12 for a discussion of immigration remedies based on trauma.

56 In the other category, respondents wrote in seeking or providing mental health forensic assessments in motions to reopen, bond hearings, Franco hearings, parole, competency assessments, and hardship waivers.

57 Between 2020 and 2022, 93,778; 63,074; and 238,841 asylum seekers filed for relief affirmatively, while 196,911; 88,162; and 253,524 filed for asylum defensively while in removal proceedings. *See* Gibson, *supra* note 53, at 9 tbls.6a & 6b.

58 U.S. Citizenship & Immigr. Servs., Characteristics of T Nonimmigrant Status (T Visa) Applicants Fact Sheet 1 (2022) (reporting that since 2008, USCIS has received a total of 30,900 T visa applications with the highest annual filing occurring in 2022 at 4,940). T visas are available to victims of human trafficking and—in addition to being numerically capped at 5,000 per year—are under-utilized both out of victims’ fear of retaliation but also due to legal impediments. *See* Julie Dahlstrom, *Trafficking and the Shallow State*, 12 U.C. Irvine L. Rev. 61, 65–67 (2021); Elisha A. Novak, Teresa C. Kulig & Cristián Doña-Reveco, *Human Trafficking and the T Visa Process: Insights from Legal Representatives Working with Victims from Latin America* 2–3 (Univ. of Neb. At Omaha, Off. of Latino/Latin Am. Stud., OLLAS Rep. No. 15, 2021), https://digitalcommons.unomaha.edu/latinamstudies_ollas_reports/17/.

59 U.S. Citizenship & Immigr. Servs., Number of Form I-918 Petitions for U Nonimmigrant Status 1 (2022) (reporting a range of 6,850 to 37,287 U visa applications received annually for years between 2009 to 2021).

60 Laurie A. Minter, *Victimization or Deportation? Addressing the Unsettling Consequences of the U Visa Requirements on Domestic Violence Victims*, 41 T. Jefferson L. Rev. 61, 73–75 (2018).

61 Meaghan Fitzpatrick & Leslye E. Orloff, *Abused, Abandoned, or Neglected: Legal Options for Recent Immigrant Women and Girls*, 4 Penn. St. J.L. & Int’l Aff. 614, 625 (2016).

62 Patrick J. Glen, In Re L-A-C-*: A Pragmatic Approach to the Burden of Proof and Corroborating Evidence in Asylum Proceedings*, 35 Geo. Immigr. L.J. 1, 8–9 (2020).

63 Trauma rarely needs to be documented in naturalization cases. One scenario in which the existence of trauma is relevant is when an applicant has mental impairments, which can include PTSD and other forms of trauma, and requests a waiver of the English language and civics examination requirements based on that impairment. *See* Emily Benson Chatzky, Note, *Almost Citizens: How the Medical Exception Continues to Limit Refugee Naturalization*, 12 Elon L. Rev. 137, 155–56 (2020) (discussing Form N-648 Medical Certification for Disability Exception). A second scenario is where an applicant struggles with drug or alcohol addiction, and therefore faces disqualification from naturalization due to the good moral character requirement. Practitioners have been creative in arguing that addiction can be a trauma response and should be considered a medical condition, not triggering inadmissibility. *See* Michelle Lough et al., Immigr. Legal Res. Ctr., Immigrants and Substance Use Disorders: A Legal and Medical Perspective, Part II: Applying Medical Evidence and Expertise on SUDs to Immigration Cases 5, 9–10 (2023).

64 Cecilia Olavarria & Moira Fisher Preda, *Additional Remedies under VAWA: Battered Spouse Waiver*, *in* Breaking Barriers: A Complete Guide to Legal Rights and Resources for Battered Immigrants 3 (Kathleen Sullivan & Leslye Orloff eds., 2013) (explaining that during the removal of conditional status, an applicant may seek a waiver to the joint filing if she was battered or the target of extreme cruelty during her marriage).

65 The other cited factors provided by lawyers for seeking mental health forensic assessments include, in rank order: failure to appear or other irregularity in the immigration proceedings (48.44%); competency concerns (46.88%); need to explain missing deadlines (46.88%); request for evidence or notice of intent to deny (38.58%); need to address potential grounds of inadmissibility (38.54%); and other (10.42%).

66
*See* Jun Du et al., *Post-Traumatic Stress Disorder: A Psychiatric Disorder Requiring Urgent Attention*, 2 Med. Rev. 219, 219 (2022) (“Although [Traumatic Events] TEs are a major risk factor for PTSD, not every victim develops PTSD after experiencing TEs.”); *see also generally* Isaac Galatzer-Levy et al., *Utilization of Machine Learning for Prediction of Post-Traumatic Stress: A Re-examination of Cortisol in the Prediction and Pathways to Non-remitting PTSD*, 7 Translational Psychiatry art. no. e1070 (2017) (utilizing machine learning methods to isolate biological risk factors for PTSD).

67
*See* sources cited *supra* note 62.

68 Arastu, *supra* note 14, at 107–08; Kate Aschenbrenner, *Ripples Against the Other Shore: The Impact of Trauma Exposure on the Immigration Process Through Adjudicators*, 19 Mich. J. Race & L. 54, 63, 67, 73 (2013).

69
*See* Arastu, *supra* note 14, at 55, 70 (discussing the role of psychological illness in the exercise of “administrative grace” and, in the context of T visas, proving presence due to trafficking).

70 Melanie A. Conroy, *Real Bias: How REAL ID’s Credibility and Corroboration Requirements Impair Sexual Minority Asylum Applicants*, 24 Berkeley J. Gender L. & Just. 1, 26–27 (2009).

71 Jeremy A. Blumenthal, *A Wipe of the Hands, A Lick of the Lips: The Validity of Demeanor Evidence in Assessing Witness Credibility*, 72 Neb. L. Rev. 1157, 1164–65 (1993) (contrasting the expansive legal dictionary definition of “demeanor” with the lay definition, which focuses on outward behavior and manner, and arguing that the lay definition is used by jurors and courts in practice); Schroeder, *supra* note 7, at 328 (“The Court of Appeals for the Eleventh Circuit has acknowledged that the lack of any statutory or regulatory definition of the term [‘demeanor’] has led to the use of ‘stereotypes about how persons belonging to a particular group would act, sound, or appear’ as ‘a substitute for [real] evidence.’”) (quoting Todorovic v. U.S. Att’y Gen., 621 F.3d 1318, 1324–27 (11th Cir. 2010)); Liz Bradley & Hillary Farber, *Virtually Incredible: Rethinking Deference to Demeanor When Assessing Credibility in Asylum Cases Conducted by Video Teleconference*, 36 Geo. Immigr. L.J. 515, 539–40 (2022) (observing that the “undefined scope of demeanor” exacerbates the problem of implicit bias and cultural misunderstanding in immigration adjudications); *see also* Miron Zuckerman, Bella M. DePaulo & Robert Rosenthal, *Verbal and Nonverbal Communication of Deception*, 14 Advances Experimental Soc. Psych. 1, 39 (1982).

72
*See* Scott Rempell, *Gauging Credibility in Immigration Proceedings: Immaterial Inconsistencies, Demeanor, and the Rule of Reason*, 25 Geo. Immigr. L.J. 377, 395–403 (2011) (analyzing the broad range of ways immigration judges can and do use demeanor in determining credibility under the REAL ID Act and the varying standards of review of demeanor findings potentially applicable on appeal); Schroeder, *supra* note 7, at 328–30 (observing that the “demeanor” component of the credibility determination is ill-defined or overly broad and partially attributing “the wide variance in asylum grant rates throughout the country … to the high level of subjectivity involved in credibility determinations”); Bradley & Farber, *supra* note 71, at 530–31 (providing examples of “adverse demeanor findings based on an applicant being ‘agitated,’ ‘visibly nervous,’ or when their speech becomes ‘notably faster’ or has an ‘almost a desperate tone’ when responding to difficult questions. Courts can point to a ‘lack of emotion’ when discussing traumatic events like incarcerations and beatings to support an adverse credibility finding. Lack of eye contact can be a basis for an adverse credibility finding”).

73 Tim Brennen & Svein Magnussen, *The Science of Lie Detection by Verbal Cues: What Are the Prospects for Its Practical Applicability?*, 13 Frontiers Psych art. no. 835285, at 1 (2022); Timothy R. Levine, *Content, Context, Cues, and Demeanor in Deception Detection*, 13 Frontiers Psych. art. no. 988040, at 2 (2022); Paul J. Taylor, Samuel Larner, Stacey M. Conchie & Sophie Van Der Zee, *Cross-Cultural Deception Detection*, *in* Detecting Deception: Current Challenges and Cognitive Approaches 175, 176 (Pär Anders Granhag et al. eds., 2015); Daniel J. Procaccini, Note, *What We Have Here is a Failure to Communicate: An Approach for Evaluating Credibility in America’s Multilingual Courtrooms*, 31 B.C. Third World L.J. 163, 177–78 (2011); Joseph W. Rand, *The Demeanor Gap: Race, Lie Detection, and the Jury*, 33 Conn. L. Rev. 1, 14 (2000); Bradley & Farber, *supra* note 71, at 534–43.

74 Kathleen T. Brady & Sudie E. Back, *Childhood Trauma, Posttraumatic Stress Disorder, and Alcohol Dependence*, 34 Alcohol Rsch. 408, 409 (2012).

75
*See* Rebecca Sharpless, *Addiction-Informed Immigration Reform*, 94 Wash. L. Rev. 1891, 1924–28 (2019) (discussing how drug addiction is viewed as an indication of moral failing in immigration proceedings and become grounds for removal).

76 Istanbul Protocol, *supra* note 12.

77 Alicia Rhodes, *Understanding Malingering: Deceptive Behavior in Psychology*, Listen Hard (Feb. 4, 2024), https://listen-hard.com/psychological-research-and-methodology/understanding-malingering-deceptive-behavior-in-psychology/ [https://perma.cc/8KP5-NT5G]. Other terms that are distinct from credibility but that could be confused are fictitious disorder and conversion disorder. Fictitious disorder involves intentional feigning of symptoms but not based on the presence of external rewards. Individuals with conversion disorder experience real physical symptoms, such as paralysis or numbness, without any conscious control over them, often in response to psychological stressors. Usually, malingering is more associated with credibility assessments since fraud or intentional deception to gain an immigration benefit (such as asylum) or to be granted relief from deportation is present in immigration adjudication.

78
*See* Schroeder, *supra* note 7, at 325–26.

79
*Id.*; *see generally* James P. Eyster, *Searching for the Key in the Wrong Place: Why “Common Sense” Credibility Rules Consistently Harm Refugees*, 30 B.U. Int’l L.J. 1, 3 (2012) (analyzing the evolution of credibility determinations and arguing that judges rely on “mechanical credibility determinations” because doing so is much easier than “looking for answers [] in the confusing and challenging area of legal rights to asylum where the truth lies”).

80
*See, e.g.*, Majse Lind, Jonathan M. Adler & Lee Anna Clark, *Narrative Identity and Personality Disorder: an Empirical and Conceptual Review*, 22 Current Psychiatry Reps. art. no. 67 (2020) (reviewing literature on the association between personality disorders and key aspects of narrative identity, including ability to conduct autobiographical reasoning and structure coherent narratives); *see also* Evert Bloemen, Erick Vloeberghs & Celine Smits, *Psychological and Psychiatric Aspects of Recounting Traumatic Events by Asylum Seekers*, *in* CARE FULL: Medico-Legal Reports and the Istanbul Protocol in Asylum Procedures 42, 55–59 (René Bruin et al. eds., 2006) (discussing how psychiatric syndromes stemming from traumatic experiences can affect how asylum seekers tell their stories); Schönfeld & Ehlers, *supra* note 11, at 326 (reporting interference of PTSD with positive autobiographical memories).

81 Ariel Washington & Jill Randall, *“We’re Not Taken Seriously”: Describing the Experiences of Perceived Discrimination in Medical Settings for Black Women*, 10 J. Racial & Ethnic Health Disparities 883, 888 (2023).

82 Dimitri Marques Abramov & Paulo-de-Tarso de Castro Peixoto, *Does Contemporary Western Culture Play a Role in Mental Disorders*, 13 Frontiers Psychiatry art. no. 978860, at 2 (2022); Narayan Gopalkrishnan, *Cultural Diversity and Mental Health: Considerations for Policy and Practice*, 6 Frontiers Pub. Health art. no. 179, at 2 (2018).

83
*See* Narayan Gopalkrishnan, *supra* note 82, at 2 (describing a study in which Afghan refugees viewed physical and mental health as one, not differentiating the two according to Western standards); *see also* U.S. Pub. Health Serv., Mental Health: Culture, Race, and Ethnicity—A Supplement to Mental Health: A Report of the Surgeon General 11 (2001) (explaining the concepts of “idioms of distress” and “culture-bound syndromes”).

84
*See* Substance Abuse & Mental Health Servs. Admin., HHS Pub. No. (SMA) 14-4816, Treatment Improvement Protocol No. 57, Trauma-Informed Care in Behavioral Health Services, 92–94 (2014) (describing protocols for screening and assessing trauma).

85
*See* Istanbul Protocol, *supra* note 12, ¶¶ 342–53.

86
*See id.* ¶ 388.

87
*See supra* notes 74–80 and accompanying text.

88 Hope Ferdowsian, Katherine McKenzie & Amy Zeidan, *Asylum Medicine: Standard and Best Practices*, 21 Health & Hum. Rts. J. 215, 216–17 (2019); *The Global Standard for Torture Documentation and Investigation*, Physicians for Hum. Rts., https://phr.org/issues/torture/setting-anti-torture-norms/istanbul-protocol/ [https://perma.cc/9ZVF-8GDC] (last visited Feb. 26, 2024).

89 Juan E. Méndez, Keynote Address at the Cornell Law School Conference: Bridging the Scientific-Legal Divide in the Treatment of Trauma in Immigration Conference (Sept. 23, 2022) https://compassioninimmigration.faculty.ucdavis.edu/news-updates/conference-bridging-the-scientific-legal-divide-in-the-treatment-of-trauma-in-immigration/ [https://perma.cc/89ZA-2GC7].

90 Notes taken during conference remarks, on file with authors. *See also* NOTES: Bridging the Scientific-Legal Divide in the Treatment of Trauma in Immigration 12 (2022), https://compassioninimmigration.faculty.ucdavis.edu/wp-content/uploads/sites/678/2023/01/COMPILED-SEPT-23-CONF-NOTES.pdf.

91 Participants were asked to select all that applied. “Number of cases” refers to the number of respondents (N=66), and “Total Response Count” refers to the cumulative number of items selected across all respondents. “Frequency” is the count of participants who endorsed each specific item. “Percent of Responses” is the percent of all endorsements that item accounts for, and “Percent of Cases” is the percent of respondents who endorsed that item.

92 Real ID Act of 2005, Pub. L. No. 109-13, 119 Stat. 231 (codified as amended at scattered sections of 8 U.S.C. and 49 U.S.C. § 30301).

93 8 U.S.C. § 1158 (b)(1)(B)(iii).

94 The authors are presently surveying Board of Immigration Appeals and Federal Circuit Court cases on appeal for adverse credibility determinations that included medical or mental health assessments to analyze how these might have impacted case outcomes, since reversal of lower court findings is much harder and can only occur if the findings were unreasonable. *See* Nicholas Narbutas, Note, *The Ring of Truth: Demeanor and Due Process in U.S. Asylum Law*, 50 Colum. Hum. Rts. L. Rev. 348, 355–56 (2018) (explaining that reviewing courts strictly apply the “substantial evidence” standard of review, under which “the IJ’s findings will only be reversed if the evidence would compel any reasonable factfinder to reach a different conclusion”).

95
*See supra* Part II.E.

96 Only 16.67% reported having no concerns, while another 8.33% were unsure.

97
*Cf.* 8 U.S.C. § 1158 (b)(1)(B)(iii) (expressly permitting judges to make credibility determinations based on inconsistencies “without regard to whether [they] go[] to the heart of the applicant’s claim”).

98 Note that the total number of ratings used to calculate each mean (sd) varies slightly based on the number of respondents who chose “N/A” for each option and were therefore not included in the calculation of that average.

99 Average ratings that differ significantly between groups are indicated with an asterisk.

100 Ratings were compared using Welch’s two-sample t-test. *See, e.g.*, Robert M. West, *Best Practice in Statistics: Use the Welch t-test When Testing the Difference Between Two Groups*, 58 Annals Clinical Biochem. 267 (2021). P-values were corrected (*p_cor_*) for 13 comparisons using the Benjamini-Hochberg procedure. *See* Yoav Benjamini & Yosef Hochberg, 57 *Controlling the False Discovery Rate: A Practical and Powerful Approach to Multiple Testing*, J. Royal Stat. Soc’y 289 (1995). Degrees of freedom vary based on total N responses for each indicator. *See supra* note 94 and Section I.

101 None of the reported perceptions of indicator importance to adjudicators significantly differs between mental health evaluators (N=38) and legal professionals (N=77, # all corrected *ps* > .05).

102 Comparison of mental health evaluators’ (mh) and legal professionals’ (lp) own importance ratings to their ratings of perceived adjudicator importance: emotional/behavioral congruence with personal story, mh: *t*(58.60) = -0.33, *p_cor_* = .740; lp: *t*(142.93) = -4.04, *p_cor_* = .001; consistency with psychological/neurocognitive models of trauma, mh: *t*(42.98) = 4.12, *p_cor_* < .001; lp: *t*(116.28) = 2.10, *p_cor_* = .045; consistency with testimony by family or other witnesses, mh: *t*(56.00) = -2.81, *p_cor_* = .009; lp: *t*(133.80) = -5.05, *p_cor_* <.001; factual consistency in personal story, mh: *t*(58.33) = -6.54, *p_cor_* < .001; lp: *t*(113.74) = -6.41, *p_cor_* < .001; temporal consistency in sequence of events, mh: *t*(60.00) = -6.36, *p_cor_* < .001; lp: *t*(121.58) = -7.20, *p_cor_* < .001; validity scales, mh: *t*(35.04) = -0.84, *p_cor_* = .443; lp: *t*(45.72) = 1.17, *p_cor_* = .250; effort testing, mh: *t*(20.97) = -0.91, *p_cor_* = .443; lp: *t*(38.60) = 1.41, *p_cor_* = .182; sufficiency of detail and specificity, mh: *t*(61.97) = -3.84, *p_cor_* < .001; lp: *t*(132.25) = -4.30, *p_cor_* < .001; plausibility, mh: *t*(52.47) = -6.87, *p_cor_* < .001; lp: *t*(117.03) = -5.19, *p_cor_* < .001; consistency across interviews/declarations/etc., mh: *t*(48.11) = -6.61, *p_cor_* < .001; lp: *t*(100.03) = -6.12, *p_cor_* < .001; demeanor, mh: *t*(59.52) = -2.56, *p_cor_* = .017; lp: *t*(142) = -4.81, *p_cor_* < .001; consistency with corroborating evidence, mh: *t*(61.56) = -4.53, *p_cor_* < .001; lp: *t*(138.29) = -4.65, *p_cor_* < .001; availability of collateral/corroborating evidence, mh: *t*(56.99) = -5.74, *p_cor_* < .001; lp: *t*(141.25) = -4.53, *p_cor_* < .001. Note that degrees of freedom vary based on N responses for that indicator. *See supra* note 94 and Section I. Methodology.

103 Istanbul Protocol, *supra* note 12, ¶ 388.

104
*Id.* ¶ 389.

105
*Id.* ¶ 546 (internal quotation marks omitted).

106
*Id.* ¶ 268.

107
*See supra* note 68 and accompanying text.

108 Mark W. Bennett, *Unspringing the Witness Memory and Demeanor Trap: What Every Judge and Juror Needs to Know about Cognitive Psychology and Witness Credibility*, 64 Am. U. L. Rev. 1331, 1365 (2015).

109 Note that the total N used for calculating each mean differs based on the number of participants who indicated “N/A” and were therefore not included in that calculation. Also note that most participants did not endorse the “other” option, thus the N for this response is significantly smaller: Mental health evaluators = 7, mental health evaluators’ perception of adjudicators = 15, legal professionals’ perception of adjudicators = 7.

110 Gail Theisen-Womersley, Trauma and Resilience Among Displaced Populations: A Sociocultural Exploration 37–38 (2006); Hannah Rogers, Simone Fox & Jane Herlihy, *The Importance of Looking Credible: The Impact of the Behavioural Sequelae of Post-Traumatic Stress Disorder on the Credibility of Asylum Seekers*, 21 Psych. Crime & L. 139, 143–48 (2015).

111 Bradley & Farber, *supra* note 71, at 536–37; *see also* Rogers, Fox & Herlihy, *supra* note 110, at 141, 151.

112 Istanbul Protocol, *supra* note 12, ¶ 497.

113
*Id.* ¶¶ 566–80, 599–600.

114 In retrospect, we probably should have included other important factors such as age or sexual orientation since these have also been shown to yield different trauma responses likely relevant to credibility. *See* Søegaard et al., *supra* note 43, at 8–9. Also, we should have asked about drug use since it is not uncommon for victims of trauma to turn to alcohol and other substance abuse as a coping mechanism. *See* Istanbul Protocol, *supra* note 12, ¶ 510. It is telling that respondents were hoping for additional categories since over 25% reported shifting their assessment of credibility completely, with another approximately 40% saying a lot by marking “other” factors without defining what these were.

115 “Other” was also highly rated, but only 7 respondents endorsed this, and of those 6 only one chose “N/A - cannot assess or do not know” so the high rating is based on a single respondent and therefore is not interpretable at a group level.

116
*See generally* César Cuauhtémoc García Hernández, Welcome the Wretched: In Defense of the “Criminal Alien” (2024) (documenting and contesting the fundamental assumption that criminal activity has a rightful place in immigration matters to determine who belongs); Juliet P. Stumpf, *Crimmigration and the Legitimacy of Immigration Law*, 65 Ariz. L. Rev. 113 (2023) (documenting how crimmigration introduces procedural deficiencies into immigration law).

117 Arastu, *supra* note 14, at 107–08.

118 Istanbul Protocol, *supra* note 12, ¶¶ 541–43.

119
*Id.* ¶¶ 497, 512–25.

120 Less relevant to issues of credibility or establishing trauma, a mental health diagnosis was found relevant by a majority or almost half of both legal and mental health professionals for: “prediction of future behavior/needs/hardship” (73.08% and 78.38%, respectively); and “to explain missed filing dates, failure to appear, or other” (56.42% and 48.65%, respectively). Many also thought a mental health diagnosis was helpful to show potential for rehabilitation: 35.90% of legal professionals and 43.24% of mental health professionals chose this category. Very few chose the category of “other” (8.97% of lawyers and 10.81% of mental health professionals).

121
*See supra* note 62.

122 As we explained, trauma can impact how memory is formed and recalled and can also influence, alongside other factors like gender and culture, how trauma is recounted. *See* John P. Wilson, *supra* note 19, at 3–4; Fino, Mema & Russo, *supra* note 20, at 2.

123 Istanbul Protocol, *supra* note 12, ¶¶ 343–353 and 387–392.

